# An integrative NLP framework identifies multilevel linguistic phenotypes of schizophrenia across tasks

**DOI:** 10.1017/S0033291726104668

**Published:** 2026-06-23

**Authors:** Hironobu Nakamura, Yoshinobu Kano, Genichi Sugihara, Ryo Takemura, Yusei Yamaguchi, Masaaki Shimizu, Shunsuke Takagi, Mari Iizuka, Saaya Tashiro, Momoko Kitazawa, Ayako Sento, Hidehiko Takahashi, Kishimoto Taishiro

**Affiliations:** 1 Department of Psychiatry and Behavioral Sciences, Institute of Science Tokyo, Japan; 2 Faculty of Informatics, Shizuoka University, Shizuoka Daigaku, Japan; 3 Clinical and Translational Research Center, Keio University Hospital, Keio University: Keio Gijuku Daigaku, Japan; 4 Department of Neuropsychiatry, Keio University School of Medicine, Keio University: Keio Gijuku Daigaku, Japan; 5 Institute of Science Tokyo School of Medicine, Institute of Science Tokyo, Japan; 6 Center for Promotion of Interdisciplinary Research in Medicine and Life Science, Keio University School of Medicine, Keio University: Keio Gijuku Daigaku, Japan

**Keywords:** computational psychiatry, diagnosis, language, natural language processing, schizophrenia

## Abstract

**Background:**

Linguistic abnormalities in schizophrenia (SCZ) span morphological, syntactic, semantic, and discourse levels. Converging cross-linguistic evidence suggests that SCZ may involve semantic narrowing alongside reduced syntactic differentiation, yet how these changes co-occur across linguistic domains and whether they represent core, task-general disturbances remains unclear. We applied a multilevel NLP framework to a large Japanese dataset to identify structurally related linguistic markers of SCZ across elicitation contexts.

**Methods:**

Speech from 104 patients with SCZ and 101 healthy controls was collected through semi-structured interviews. Transcripts from free conversation, storytelling, and picture description were analyzed using GiNZA, Word2Vec, TF-IDF, and SentenceBERT to extract 76 morphosyntactic, semantic, and discourse features. Factor analysis identified representative features independent of diagnosis, which were tested using generalized estimating equations and validated with bootstrap and permutation procedures. Cross-task stability was examined to determine core linguistic markers.

**Results:**

In free conversation, reduced Case-particle (Kakujoshi) and Adverb use and increased Mean Pairwise Word Similarity were strongly associated with SCZ (AUC = 0.87, 95% CI: 0.74–0.97). Adverbial, case-particle, and semantic-network measures functioned as cross-task markers.

**Conclusions:**

SCZ involves multidimensional language disturbances characterized by a tripartite linguistic phenotype of diminished morphosyntactic explicitness, semantic narrowing, and reduced modification-based contextual modulation in spontaneous discourse. Extending cross-linguistic evidence, our results indicate that lexical-semantic contraction co-occurs with reduced overt marking of argument relations in Japanese, alongside weakened adverbial elaboration and framing – suggesting convergent, largely task-general dimensions of SCZ language pathology, most evident in free conversation.

## Introduction

Linguistic abnormalities in the speech of individuals with schizophrenia (SCZ) have long been studied (Andreason & Grove, 1986; Corcoran & Cecchi, 2020; Covington et al., 2005; Ehlen, Montag, Leopold, & Heinz, 2023; Hinzen & Rosselló, 2015; Kuperberg, 2010a; Kuperberg, 2010b; Spitzer, 1993). In recent years, NLP has been increasingly employed to quantify these abnormalities (Chan et al., 2023; Corcoran et al., 2020; Corcoran & Cecchi, 2020; Fradkin, Nour, & Dolan, 2023; Murphy & Öngür, 2022; Sarzynska-Wawer et al., 2021; Srivastava et al., 2023). NLP has shown potential clinical applications, including detection (Irving et al., 2021), monitoring of treatment response (Chekroud et al., 2021) and illness course (Dalal et al., 2025), prediction of psychosis onset (Irving et al., 2021; Silva et al., 2023), and relapse (Birnbaum et al., 2019). Earlier NLP studies have examined linguistic abnormalities in SCZ across multiple levels of analysis – including morphological, syntactic, semantic, and discourse domains – reporting impairments in semantic coherence (Corcoran et al., 2018; Nour, McNamee, Liu, & Dolan, 2023), syntactic complexity (de Boer et al., 2020), poverty of content (Rezaii, Walker, & Wolff, 2019), referential cohesion (Lundin et al., 2020), and related features (Bambini et al., 2022; Gutierrez, Cecchi, Corcoran, & Corlett, 2017).

Several computational models quantify linguistic abnormalities in SCZ. For example, Mota et al. (2012) introduced the ‘speech graph’ approach, representing words as nodes linked by temporal succession. Graph properties such as connectedness and recurrence quantify structural disorganization in psychosis and have been associated with positive thought disorder severity. Positive FTD has also been analyzed using latent semantic analysis (LSA) (Elvevåg, Foltz, Weinberger, & Goldberg, 2007) and coherence models (Bedi et al., 2015; Just et al., 2019), which evaluate semantic associations by converting words or sentences into vectors. In contrast, negative FTD, characterized by poverty of speech and reduced ideational content, has been studied using the semantic density model (Rezaii et al., 2019).

NLP methods, including the models mentioned above, have been utilized at various levels of linguistic analysis. At the morphological level, researchers have examined features such as part-of-speech distribution (Morgan et al., 2021), pronoun frequency (Bambini et al., 2022), word count, and word length. At the syntactic level, measures of dependency structure and complexity, including tree depth (Corcoran & Cecchi, 2020), have been employed. At the semantic level, studies have focused on anomalies in word or sentence meaning, such as repetition (Tang et al., 2021) or ambiguous pronouns, often without considering contextual information. Furthermore, recent work has highlighted increased local semantic proximity, suggesting constrained semantic exploration in SCZ and a shift in the geometry of semantic space (Arslan et al., 2024; Palominos et al., 2025; Pintos et al., 2022). At the discourse level, coherence and tangentiality have primarily been assessed using vector-based representations. Together, these approaches yield a wide range of features that reflect the hierarchical organization of language processing, from lower-level lexical operations to higher-order discourse management.

Despite this breadth, most prior studies – apart from a few multitask investigations (Mota et al., 2023; Tang et al., 2021) – applied features in isolation within small, single-task samples, limiting their ability to capture context-dependent language variation. In addition, the range of linguistic features examined was often insufficient to reflect the multidimensional structure of language. Although recent work has proposed composite linguistic indices to address multidimensionality (Palominos et al., 2025), we argue that preserving interpretability requires identifying task-general features across linguistic levels rather than collapsing disturbances into a single summary score.

To address this gap, we adopted a multilevel framework that reflects the hierarchical organization of language. By systematically sampling features across morphological, syntactic, semantic, and discourse domains, we sought to capture the multidimensional structure of language disturbance in SCZ. To identify latent structures, we retained the multidimensional structure and focused on representative features within each latent domain to preserve interpretability. This strategy enables a principled reduction of complexity without obscuring the distinct linguistic processes that contribute to language pathology.

We then conducted a comprehensive NLP study using large patient samples with SCZ and HCs across three tasks. Based on prior theoretical and empirical work suggesting that SCZ links to reduced grammatical elaboration and constrained semantic retrieval (Corcoran & Cecchi, 2020; Covington et al., 2005; Kuperberg, 2010a; Spitzer, 1993), we hypothesized that two core linguistic abnormalities would distinguish patients from controls: reduced syntactic differentiation and increased semantic proximity (i.e. more restricted word-to-word semantic associations). To test these hypotheses, we combined hypothesis-driven and data-driven methods within this multilevel framework. This design bridges classical constructs with modern computational tools, enabling more comprehensive characterization of language pathology in SCZ across tasks.

## Materials and methods

### Participants and procedures

This study was conducted within the Understanding Psychiatric Illness Through Natural Language Processing (UNDERPIN) project (protocol: Kishimoto et al., 2022), which enrolls participants with DSM-5/ICD-10 diagnoses of major depressive disorder, bipolar disorder, schizophrenia, anxiety disorders (including obsessive-compulsive disorder), or mild cognitive impairment/dementia. In the present analysis, the SCZ group comprised participants with SCZ without other UNDERPIN target diagnoses; other psychiatric and medical conditions were not controlled beyond the predefined exclusion criteria. The sample included 104 patients with SCZ and 101 HCs, recruited from 7 hospitals and 3 outpatient clinics in Japan between 2018 and 2024. Patients were aged 20 years or older and met DSM-5 or ICD-10 criteria for SCZ. HCs were adults without any lifetime history of psychiatric disorders. All participants were fluent in Japanese and provided written informed consent after a full explanation of the study. Patients were excluded if participation was judged by the treating psychiatrist to impose a substantial clinical burden or if medical or neurological conditions could interfere with speech recording. Healthy controls were excluded if they had medical or neurological conditions affecting speech production. Detailed inclusion and exclusion criteria are provided in the Supplementary Materials. The study was approved by the institutional review boards of Keio University School of Medicine and participating institutions. Each participant completed two to five visits at ≥1-month intervals to capture within-subject variability and enhance reliability while minimizing practice effects. During each visit, speech samples were collected through three semi-structured tasks: (1) free conversation, (2) storytelling, and (3) picture description. Free conversation, covering everyday topics such as routines and personal interests, assessed spontaneous speech production with minimal external constraints. Storytelling, using either Cinderella or the Japanese folktale Kaguya-hime (if the former was unfamiliar), assessed narrative planning, temporal sequencing, and discourse coherence. Picture description, using standardized visual stimuli from the Visual Perception Test for Agnosia (VPTA), the Neurobehavioral Cognitive Status Examination (COGNISTAT), and the Western Aphasia Battery (WAB), required participants to generate speech guided by structured visual input, thereby taxing lexical retrieval and descriptive organization. Each 30–60-minute session was recorded using dual headset microphones to ensure audio quality and speaker separation. Interviewers maintained a neutral style and minimized interventions to reduce examiner influence on speech output. The severity of illness was assessed with the Positive and Negative Syndrome Scale (PANSS).

### Speech data processing and feature extraction

All recorded speech data were transcribed into text and processed to extract only the participant’s speech. All speech data were transcribed verbatim by a professional service without normalization or removal of disfluencies. Manually inserted punctuation defined sentence boundaries for sentence-distance analyses, whereas dependency parsing was performed after removing punctuation and letting GiNZA determine sentence boundaries automatically, ensuring independence from manual segmentation.

Linguistic features were derived from three complementary levels, each targeting distinct aspects of language processing:

1. Morphological and syntactic features using GiNZA (Matsuda, 2020; Nivre et al., 2016; Nivre et al., 2020), a Japanese parser based on Universal Dependencies (UD), which provides standardized part-of-speech tagging and dependency parsing to quantify grammatical structure and complexity;
2. Semantic features using Word2Vec (Mikolov, Chen, Corrado, & Dean, 2013; Suzuki, 2018) and Term Frequency–Inverse Document Frequency (TF-IDF) (Sparck Jones, 1972) to characterize semantic associations and network properties of word usage (Sasahara, 2015); and
3. Discourse features using SentenceBERT (Reimers & Gurevych, 2019; Sonoisa, 2020), a transformer-based model that generates contextualized sentence embeddings, enabling measurement of sentence-to-sentence similarity and discourse coherence.

(We used a publicly available Japanese Word2Vec model trained on Wikipedia to enhance transparency and facilitate interpretation of relative group differences.)

In total, 76 features were calculated per task and per visit, covering lexical, syntactic, semantic, and discourse aspects. Feature definitions and formulas are detailed in the Supplementary Method and Supplementary Table S1. When necessary, features were normalized by the Number of Morphemes to account for differences in utterance length.

### Data partitioning and preprocessing

To prevent data leakage and ensure unbiased model evaluation, all visits from the same participant were treated as a single unit during data partitioning. The dataset (104 SCZ and 101 HCs) was randomly split at the participant level into a training set (80%) and a test set (20%), stratified by diagnosis, sex, and age group to maintain comparable distributions across splits.

All feature selection and model estimation were performed solely on the training set. To account for differences in utterance length, features were expressed as ratios where appropriate and standardized using parameters derived from the training data. Features with zero values across all participants were removed. As missing values were rare (on average 0.22% in Task 1 and < 0.01% in Tasks 2 and 3), missing entries were imputed using the overall mean (see in detail Supplementary Materials S1–S2).

### Statistical analyses

**Figure 1.**
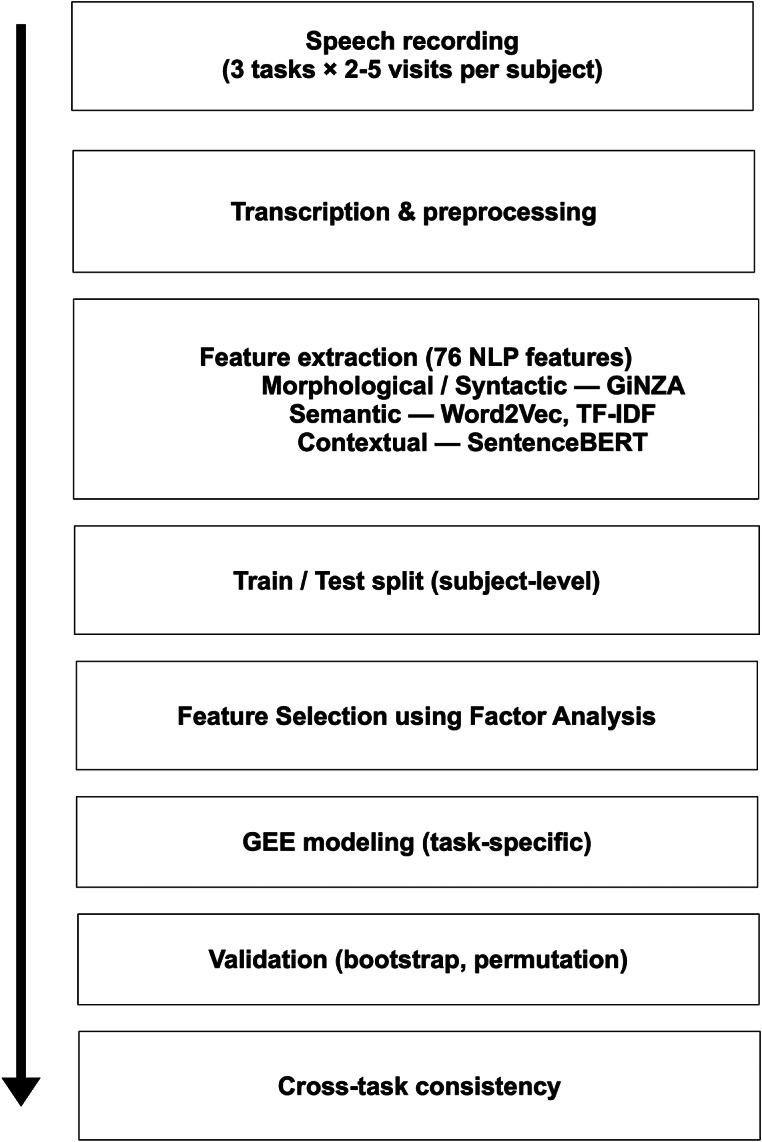
**Overview of the analysis pipeline.** A schematic illustration of the whole workflow, including: speech recording, manual transcription, extraction of 76 linguistic features using NLP, training–test data split, preprocessing, exploratory factor analysis, generalized estimating equation (GEE) modeling, model validation, and cross-task consistency assessment using partial-conjunction tests.

All analyses followed a unified pipeline (Figure 1), proceeding through four stages: (a) exploratory group comparisons, (b) dimensionality reduction, (c) multivariate modeling, and (d) cross-task validation. All thresholds, seeds, and decision rules were prespecified and archived in a machine-readable summary.

### Exploratory group comparisons

For each task, exploratory group comparisons were performed using univariate generalized estimating equations (GEEs) with robust (sandwich) standard errors to account for the correlation induced by repeated visits within participants. Age and sex were included as covariates. *P*-values were corrected for multiple comparisons using the Benjamini–Hochberg false discovery rate (FDR; *q* < 0.05).

### Dimensionality reduction via exploratory factor analysis

To address the high dimensionality and inter correlation among the 76 linguistic features, we applied exploratory factor analysis (EFA) to subject-averaged, z-standardized features. The number of factors was determined using Horn’s parallel analysis, and the resulting solution was estimated with a common-factor model and Varimax rotation. To enhance interpretability and avoid factor-score indeterminacy, we selected a single representative feature per factor (McNeish and Wolf, 2020). Features were chosen based on high primary loading, a sufficient loading gap, and low redundancy with already selected representatives. When no feature met all thresholds, predefined relaxation rules were applied to ensure that each factor yielded one representative feature. Model adequacy was examined through residual correlations between observed and reproduced matrices, and robustness was evaluated via bootstrap and sub sampling procedures with Procrustes alignment. We report Tucker’s congruence indices and the selection frequencies of representative features.

### Multivariate modeling

Task-specific GEE models were fitted to examine associations between SCZ diagnosis and the representative features, adjusting for age and sex. Models used a logit link and participant-clustered robust standard errors. Odds ratios with 95% CIs were reported, and p-values were FDR-adjusted across predictors. Predictive performance was evaluated in the held-out test set using AUC and threshold-dependent sensitivity and specificity. Confidence intervals were estimated via patient-level bootstrap, and an empirical null distribution of AUC was obtained through stratified permutation testing.

### Cross-task consistency analysis

Cross-task replication was assessed using the partial-conjunction (PC) framework, testing whether ≥2 of 3 tasks showed a significant diagnostic effect for each feature. PC *p*-values were computed from the second-order statistic of task-specific *p*-values using a conservative combinatorial bound and corrected using BH-FDR (*q*_PC_ < 0.05). As a sensitivity analysis, per-feature GEE models including a Group × Task interaction were fitted to examine heterogeneity, and Wald *p*-values were summarized descriptively.

### Software

GEEs, bootstrapping, and permutation testing were implemented in Stata 17.0. All other analyses – including preprocessing and factor analysis – were conducted in Python using libraries (scikit-learn (Pedregosa et al., 2011), statsmodels (Seabold & Perktold, 2010), scipy (Virtanen et al., 2020), and pingouin (Vallat, 2018).

## Results

### Demographics

Demographics are summarized in Table 1. SCZ patients and HCs did not differ significantly in age or gender distribution.

**Table 1.** Demographic and clinical characteristics of patients with SCZ (SCZ) and HCs (HC) Table 1. long description.

	SCZ (*n* = 104)	HC (*n* = 101)	*p*-value
Age (years)	48.3 ± 12.7	51.3 ± 18.1	0.166
Gender (female), *n* (%)	50 (48.1%)	56 (55.4%)	0.360
PANSS total	63.3 ± 18.0	30.7 ± 1.3	< 0.001
PANSS positive	15.4 ± 6.0	7.2 ± 0.5	< 0.001
PANSS negative	16.4 ± 6.3	7.3 ± 0.4	< 0.001
PANSS general psychopathology	31.4 ± 8.6	16.3 ± 0.7	< 0.001
Visits per participant (median [IQR])	2 [1–4]	2 [2–2]	0.096
Task participation – Free conversation (Task 1)	104	101	-
Task participation – Storytelling (Task 2)	101	100	-
Task participation – Picture description (Task 3)	102	101	-
Education (years)	13.3 ± 2.4 (N = 51)	15.4 ± 2.8 (N = 70)	< 0.001

*Note:* Values are mean ± SD unless otherwise indicated. Education years were available for a subset (SCZ: *N* = 51, HC: *N* = 70).

### NLP features

We analyzed 76 NLP-derived features spanning multiple linguistic domains. Feature definitions are provided in Supplementary Table S1.

### Exploratory group comparisons and correlational analysis

Exploratory analyses were initially performed to identify linguistic features that differ between SCZ and HCs in Task 1. Out of 76 NLP-derived features, 35 showed significant differences after FDR correction (*q* < 0.05), spanning morphological, syntactic, semantic, and discourse domains. As illustrated in the volcano plot (Supplementary Figure S1/S2) and summarized in Supplementary Table S3, the most distinguishing features included reduced use of Adverb and Adverbial Modifiers, as well as increased Named Entity (PERSON) Ratio and Compound Ratio, and increased Mean Pairwise Word Similarity. The increased Named Entity (PERSON) ratio is broadly consistent with prior reports of altered referential structure and increased noun phrase density in psychotic discourse (Palominos, Figueroa-Barra, & Hinzen, 2023). These features were derived from exploratory effect size rankings and do not necessarily correspond to representative features identified in the subsequent factor analysis.

Correlational analyses further revealed that linguistic features, such as Adverbial Modifiers Ratio and Mean Pairwise Sentence Distance, were significantly associated with multiple PANSS domains (Supplementary Figure S4). These exploratory results indicate widespread and consistent linguistic abnormalities across different levels of language organization, providing empirical justification for dimensionality reduction via factor analysis to identify latent constructs and reduce redundancy before multivariate modeling.

### Factor analysis and representative feature selection

Horn’s parallel analysis for Task 1 supported a nine-factor solution, which remained stable across bootstrap replications (Supplementary Figure S6 and Supplementary Tables S4–S6). After Varimax rotation, the nine factors together accounted for 62.5% of the total variance. The first factor captured the largest proportion of variance (19.6%), followed by modest but relatively even contributions from the remaining components (4.0%–7.1%).

For each factor, we selected one representative feature based on a prespecified loading criterion: the absolute loading had to be ≥0.60 and at least 0.20 larger than the next highest loading (Supplementary Tables S7–S9). This procedure yielded nine task-specific representative features (Table 2). Consistent with this dimensional reduction strategy, multicollinearity among the selected predictors was low (all VIFs <3). Detailed diagnostics are provided in Supplementary Tables S33–S35.

**Table 2.** Factor structure and representative features (Task 1) Table 2. long description.

Factor	High-loading features (examples)	Representative feature
F1	**Interjection ratio**, Discourse ratio, General Interjection ratio	Interjection ratio
F2	**Adverb ratio**, Adverbial Modifier ratio, Mean Pairwise Sentence Distance	Adverb ratio
F3	**Normalized Tree Height**, Average Sentence Length, Normalized Dependency Distance	Normalized Tree Height
F4	**Sentence-final Particle (Shuujoshi) ratio**, Particle ratio, Marker ratio	Sentence-final Particle ratio
F5	**Number of Sentences**, Redundancy (Toto et al., 2014), Vocabulary ratio	Number of Sentences
F6	**Mean Pairwise Word Similarity,** Average Distance of Semantic Graph, Variance Pairwise Word Similarity	Mean Pairwise Word Similarity
F7	**Case Particle (Kakujoshi) ratio**, Adposition ratio, Object ratio	Case Particle (Kakujoshi) ratio
F8	**Nominal Modifier ratio**, Noun ratio, Suffix (Setsubiji) ratio	Nominal Modifier ratio
F9	**Prenominal Adjective (Rentaishi) ratio**, Filler Interjection ratio, Determiner ratio	Prenomial Adjective ratio

*Note*: Horn’s parallel analysis indicated a nine-factor solution. The table shows, for each factor (F1–F9), the features with the highest loadings (examples) and the single representative feature chosen based on the largest absolute loading. These representative features were used in the subsequent GEE models. All features were standardized prior to analysis, and factor extraction was followed by Varimax rotation. Abbreviations: F, factor.

### Task 1-specific associations of representative features with SCZ diagnosis

**Figure 2.**
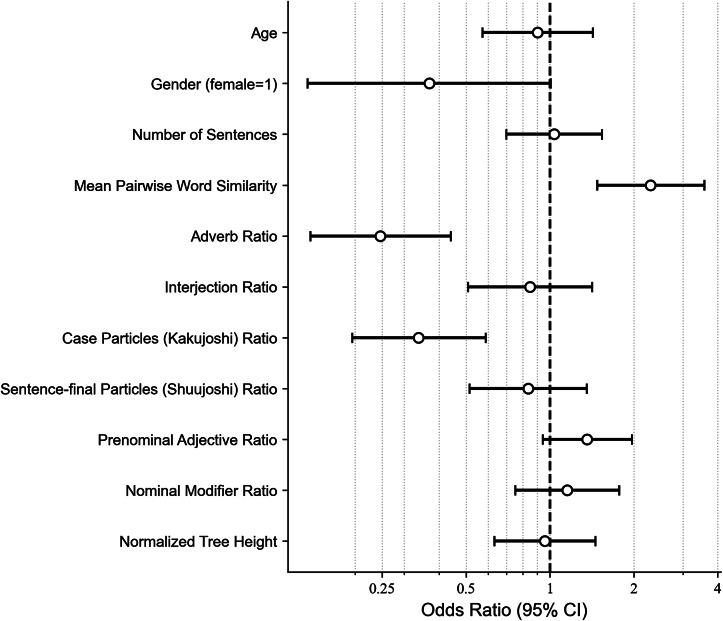
**Forest plot of odds ratios for representative linguistic features in task 1.** This plot displays the relationships between specific linguistic features and SCZ diagnosis. Odds ratios (OR) with 95% confidence intervals are shown. Significant predictors include Mean Pairwise Word Similarity, Case Particles (Kakujoshi) ratio, and Adverb ratio. Figure 2. long description.

Task 1-specific associations of representative features with SCZ diagnosis are presented in Figure 2 and Supplementary Table S19. Among the linguistic features, higher Mean Pairwise Word Similarity, lower Case Particle (Kakujoshi) Ratio, and reduced Adverb Ratio were significantly associated with SCZ diagnosis after adjusting for age and gender. For example, a 1-standard deviation increase in Mean Pairwise Word Similarity corresponded to a 2.3-fold increase in the odds of SCZ diagnosis. No other representative features showed statistically significant associations.

Education years differed significantly between groups; however, education data were available only for a subset of participants (SCZ: *N* = 51; HC: *N* = 70). Therefore, education was not included as a covariate in the primary analyses. Importantly, an exploratory complete-case sensitivity analysis adjusting for education yielded consistent results, and all three significant features remained significant; details are provided in Supplementary S28 and S29.

### Predictive performance

The GEE model demonstrated strong discriminative performance in the independent test set (AUC = 0.87, 95% CI = 0.74–0.97; permutation *p* < 0.001). Using the optimal threshold derived from the training data, the balanced accuracy was 0.83, with a sensitivity of 0.76 and a specificity of 0.90 (see also Supplementary Table S20).

### Comparison among the speech tasks

**Figure 3.**
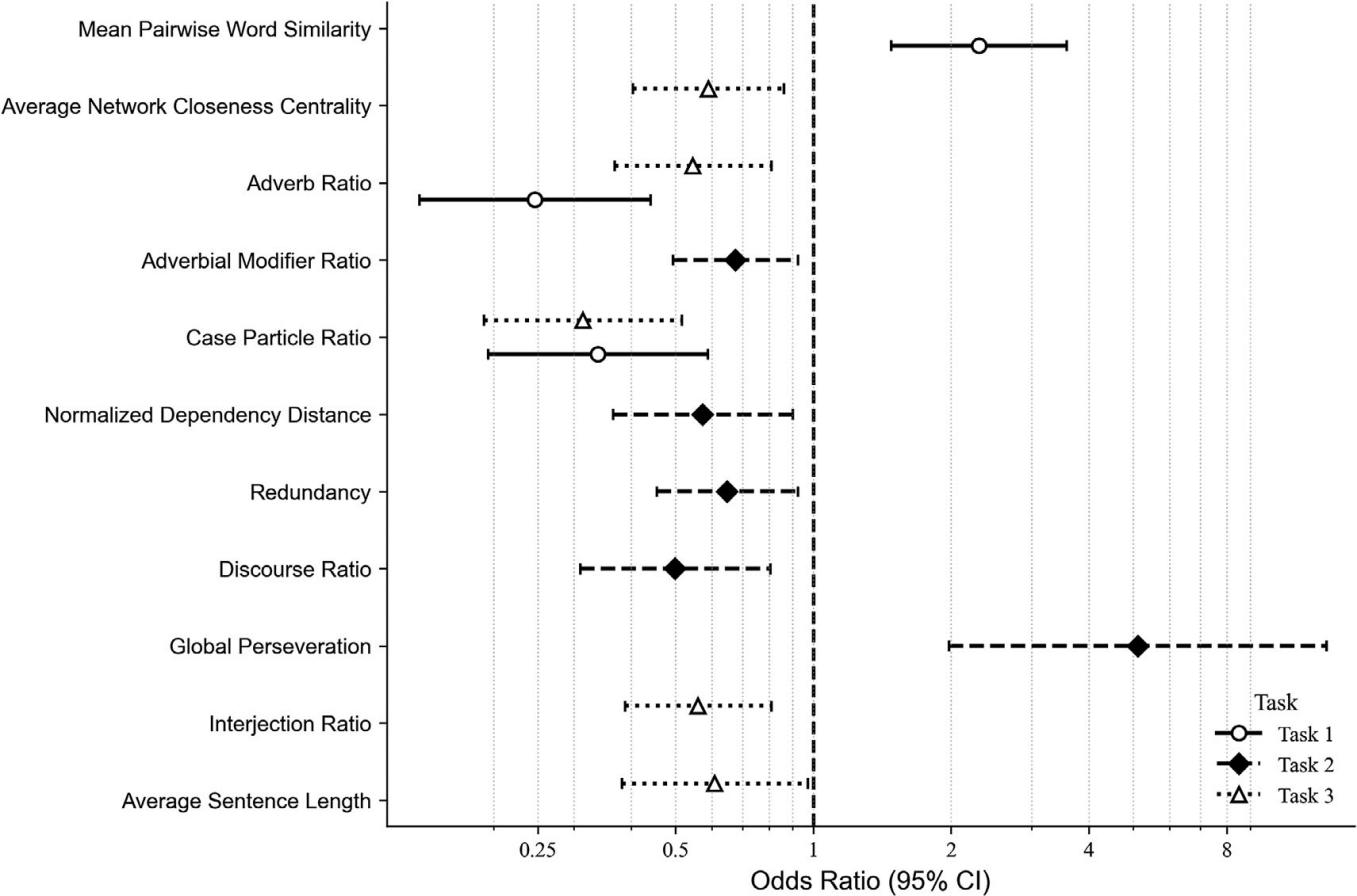
**Comparison of three speech tasks.** Features related to adverbial modification (Adverbial Modifier ratio, Adverb ratio) were strongly linked to diagnosis across all tasks. Measures of semantic networks, such as Mean Pairwise Word Similarity and Average Network Closeness Centrality, as well as Case Particle use, were significant in Tasks 1 and 3. Additionally, discourse-related features, such as Discourse ratio and Interjection ratio, were significant in Tasks 2 and 3. Overall, adverbial modification consistently served as a key diagnostic marker across tasks, while semantic network indices, case particles, and discourse features also showed relevance across different tasks. This figure summarizes three separate task-specific models; it does not imply a joint model. Figure 3. long description.

Adverbial modification features were consistently significant across all tasks, while semantic network measures, case particle use, and discourse-related features also demonstrated diagnostic relevance across several tasks (see Figure 3).

To examine whether linguistic abnormalities differed across conversational contexts, we evaluated task-specific consistency and sensitivity of representative linguistic features. Partial-conjunction analysis combining *p*-values from three task-specific GEE models revealed that both Adverb Ratio and Case Particles (Kakujoshi) Ratio showed significant cross-task consistency (*q*_PC_ = 0.021 and 0.014, respectively), indicating robust diagnostic effects across at least two of the three tasks. Sensitivity analyses including task-by-feature interactions in GEE models demonstrated that Adverb Ratio showed a significant interaction with task (Wald *χ*^2^(2) = 35.2, *p* < 0.001), with the strongest negative association in the free conversation task and attenuated effects in storytelling and picture-description tasks. In contrast, the Case Particles (Kakujoshi) Ratio remained negatively associated with diagnosis (*β* = −0.66, *p* < 0.001) without a significant task interaction, indicating a task-invariant syntactic simplification. Age and gender covariates were not significant in either model.

## Discussion

Using an integrated NLP framework in a large Japanese cohort, we identified SCZ-associated language differences that recur across elicitation contexts and linguistic levels. A representative-feature strategy (factor analysis independent of diagnosis) followed by GEE testing highlighted three strong free-conversation markers – reduced Case Particle (Kakujoshi) Ratio, increased Mean Pairwise Word Similarity, and reduced Adverb Ratio – supporting a parsimonious yet discriminative characterization of spontaneous speech disturbance. Cross-task analyses further indicated that Case marking and Adverbial modification are among the most consistently diagnosis-relevant components.

### Interpretation of core linguistic markers

The three markers associated with SCZ in Task 1 capture complementary components of disturbance that map onto distinct demands of spontaneous language production: explicit relational marking, lexical-semantic exploration, and contextual modulation.

- Case Particle Ratio

In Japanese, Case Particles (Kakujoshi) overtly encode syntactic relations and contribute to propositional interpretation by clarifying argument structure and predicate-argument links (Ito, 2002; Masuoka & Takubo, 2024). Accordingly, a reduced Case Particle Ratio suggests attenuated propositional relational explicitness – less overt marking of “who did what to whom,” and weaker structural and semantic transparency between constituents. Although particle omission can occur in everyday conversation, an SCZ-associated reduction may reflect reduced deployment of explicit relational marking under spontaneous production demands (planning, monitoring, and self-repair), potentially contributing to simpler syntactic realizations (de Boer et al., 2020). Convergently, exploratory analyses showed reductions in proposition-relevant particles (Case and Focus Particles [Keijoshi]) but not Sentence-Final Particles (Shuujoshi), suggesting greater impact on propositional grammatical marking than on pragmatic and interactional signaling (Supplementary Figure S2).

- Mean Pairwise Word Similarity

Increased Mean Pairwise Word Similarity indicates more locally clustered lexical selection in embedding space, suggesting constrained semantic exploration (i.e. selecting semantically proximate items), aligning with descriptions of reduced informational density in SCZ (Rezaii et al., 2019). At the lexical-selection level, this suggests reliance on nearby semantic neighborhoods, which may aggregate into the macro-scale network pattern of locally dense, yet globally less integrated semantic structure described below.

- Adverb Ratio

In line with previous reports (Arslan et al., 2024; Tang et al., 2021; Ziv et al., 2022), a reduced Adverb Ratio was significantly associated with SCZ and negatively correlated with PANSS scores. Notably, among major open-class parts of speech – nouns, verbs, adjectives, and adverbs – only adverbs showed a significant reduction (Supplementary Figure S2). In Japanese, Adverbs modify not only verbs but also predicates, modifiers, and even entire sentences, enriching expressions of manner, degree, tense aspect, and discourse level (Masuoka & Takubo, 2024).

Importantly, the latent factors on which Adverb Ratio and the dependency-based Adverbial Modifier Ratio showed the highest loadings also included discourse/coherence-related features (e.g. Mean Pairwise Sentence Distance and Mean Adjacent Sentence Distance). This convergence suggests that the observed reduction is not merely a morphosyntactic-level change but reflects a broader weakening of modification-based tuning of utterances. We therefore interpret reduced adverbial modification as diminished contextual modulation in a broad sense – that is, a reduced capacity to elaborate, nuance, and frame content online during spontaneous discourse – rather than as a primary impairment of propositional structure. This interpretation aligns with classical descriptions of impoverished elaboration in formal thought disorder (Covington et al., 2005; Hinzen & Rosselló, 2015; Kuperberg, 2010a; Spitzer, 1993).

Together, these disruptions outline a tripartite phenotype in free conversation: (i) reduced morphosyntactic explicitness (attenuated relational marking via Case Particles), (ii) constrained semantic exploration (locally clustered lexical selection indexed by Mean Pairwise Semantic Similarity), and (iii) diminished contextual modulation (reduced Adverb Ratio).

Although we lacked direct cognitive or social functioning measures, the core linguistic markers were significantly associated with PANSS severity (see Supplementary Figure S5). Given prior work linking symptom burden to functional and cognitive outcomes (Ventura et al., 2009), these linguistic measures may serve as accessible proxies for broader impairment. Mechanistically, reduced Case Particle Ratio could reflect increased difficulty maintaining explicit argument-structure encoding under planning and monitoring demands, whereas reduced Adverb Ratio and increased Mean Pairwise Word Similarity may reflect less flexible context-sensitive elaboration. These interpretations remain hypothetical and should be tested with concurrent cognitive and functional assessments.

### Language internal properties and typological perspective

The characteristics of the Japanese language may have influenced the present results. First, given the broad modifying role of adverbs in Japanese, reductions in adverbial usage may be particularly sensitive to declines in contextual modulation. Second, from a typological perspective, Japanese is classified as an agglutinative language, similar to Turkish. In agglutinative languages, a single word is composed of multiple morphemes that can be clearly segmented (Comrie, 1988). In other words, agglutinative morphology makes grammatical marking more discretely segmentable, potentially increasing the detectability of reductions in overt relational markers. Actually, recent NLP studies in Turkish have reported findings consistent with ours, including instability in referential structure, accompanied by reductions in grammatical markers such as determiners, and increased semantic similarity (Arslan et al., 2024; Çabuk et al., 2024; Çokal et al., 2026). The former changes result in noun phrases whose referents are less clearly identifiable. Although referential instability in Turkish and case ambiguity in Japanese are formally distinct phenomena, both may reflect attenuation of relational marking. Importantly, this convergence suggests that reduced relational explicitness may reflect shared vulnerabilities in language production across agglutinative systems, while also highlighting that typological properties can shape which disturbances are most readily detected by NLP features.

### Altered semantic geometry and FTD

In line with our theoretical prediction of a restricted semantic geometry in schizophrenia, semantic network analyses revealed increased clustering and density but decreased average path length, network diameter, and closeness centrality in patients. This configuration reflects locally dense but globally less integrated semantic networks, limiting flexible traversal across distant semantic domains. These findings converge with growing cross-linguistic evidence demonstrating a contraction or ‘shrinking’ of semantic space in schizophrenia. Computational studies across diverse languages consistently report elevated semantic similarity and reduced graph diameter, suggesting constrained navigation across distributed semantic regions (Alonso-Sánchez, Limongi, Gati, & Palaniyappan, 2023; Arslan et al., 2024; Pintos et al., 2022; Voppel et al., 2023). Furthermore, this linguistic configuration parallels neuroimaging findings of altered semantic network organization in schizophrenia, including more homogeneous within-module connectivity and reduced long-range integration (Hayashi et al., 2024; Matsumoto et al., 2023). Despite methodological differences, this convergence suggests restricted access to distributed semantic representations. Such constrained semantic traversal may contribute to the tangential, perseverative, or impoverished quality of FTD in SCZ.

### Task-specific and cross-task patterns

Task-specific features reflected the distinct cognitive demands of each elicitation paradigm, consistent with psycholinguistic models positing that different speech contexts engage different components of the language production system. Syntactic structural complexity features did not emerge as significant in the free conversation task, possibly because the unconstrained nature of the task allows speakers to avoid complex constructions, thereby masking syntactic deficits (Nettekoven et al., 2023). Conversely, in the storytelling task, reduced Normalized Dependency Distance indicated diminished syntactic structural complexity, likely reflecting the working memory demands required to plan and produce sentences with long-distance dependencies. In the picture description task, a shorter Average Sentence Length and reduced Interjection use may reflect the cognitive burden of converting visual input into verbal descriptions, as well as patients’ tendency to produce minimal or truncated utterances when guided by external stimuli. Notably, although discriminative features varied across tasks, each task demonstrated comparably high classification performance. Free conversation – when sufficient time is provided – may be particularly sensitive to the ‘semantic narrowing’ characteristic of SCZ. In contrast, picture description tasks can efficiently capture syntactic and vocabulary-related abnormalities within shorter assessment windows, as prior studies suggest (Corcoran & Cecchi, 2020; Ehlen et al., 2023; Morgan et al., 2021).

### Implications for task design in future research

Although task dependence has been noted in SCZ-language research (Corcoran & Cecchi, 2020; Ehlen et al., 2023; Morgan et al., 2021), no consensus has emerged regarding optimal paradigms. Our results suggest that tasks differ in sensitivity to linguistic domains; thus, multitask assessment is preferable. We highlight desirable task characteristics:

1. Unstructured, spontaneous generation tasks to reveal reduced elaboration and semantic geometry.
2. Paradigms that minimize world-knowledge and working-memory demands to reduce cognitive confounds that can suppress speech quantity or complexity.
3. Prompts targeting internal experiences (e.g. dreams, hallucinations, and delusions) to elicit thought-disordered speech.

Future empirical studies employing standardized tasks that incorporate these characteristics will be essential for establishing robust assessment protocols for NLP-based detection of SCZ.

The heterogeneity consistently reported in schizophrenia research suggests that language disturbance should not be conceptualized as a uniform deficit. Given the multidimensional nature of schizophrenia and speech production, variability within patient groups is expected. Multitask, multidimensional NLP approaches may facilitate data-driven subtyping of linguistic profiles in future studies.

### Multidimensional nature of language dysfunction in SCZ

Previous NLP research has primarily emphasized sentence-level syntactic abnormalities and reduced semantic coherence (Corcoran et al., 2018). Our findings extend this view by suggesting that language pathology in SCZ is better characterized as a coordinated reconfiguration across three interrelated dimensions: (i) reduced morphosyntactic explicitness, (ii) altered semantic geometry, and (iii) weakened contextual modulation. Language pathology in SCZ may therefore be conceptualized within this tripartite framework, shifting from a unitary account of FTD toward separable but interacting dimensions that extend beyond traditional positive–negative dichotomies.

## Limitations

This study has several limitations. First, the sample included only patients with established SCZ and healthy controls, excluding clinical high-risk or prodromal populations; thus, the findings reflect illness characterization rather than prediction of onset. Second, symptom severity was predominantly mild-to-moderate (mean PANSS total = 63.3), limiting generalizability to severe cases. Third, symptom assessment relied solely on the PANSS without dedicated FTD scales (e.g. TLC), precluding direct mapping between computational markers and FTD phenomenology.

Fourth, the study focused exclusively on Japanese, an agglutinative language with grammatical features distinct from analytic languages. Although results aligned with prior cross-linguistic findings, replication across typologically diverse languages is needed to separate universal from language-specific effects. Fifth, education data were incomplete and could not be included in the primary models, and direct cognitive and social functioning measures were unavailable. Sixth, our lexical-semantic measures were based on a single Japanese Word2Vec model trained on Wikipedia. Although this provided a transparent semantic reference, the findings may depend on the embedding model and training corpus and should be tested with alternative embeddings, such as fastText and transformer-based models. Finally, all data were derived from oral interviews; written language may capture partially distinct aspects of discourse organization. Future studies integrating multimodal, longitudinal, and cross-linguistic designs are warranted.

## Conclusions

This study provides a large-scale quantitative characterization of language abnormalities in Japanese-speaking patients with SCZ using a multilevel NLP framework. We identified three core markers – diminished use of case particles, altered semantic geometry, and reduced adverbial modification – that demonstrated robust diagnostic discrimination and cross-task stability. These findings highlight the clinical potential of spontaneous speech and clarify the multidimensional structure of language dysfunction in SCZ.

## Supporting information

10.1017/S0033291726104668.sm001 Nakamura et al. supplementary material Nakamura et al. supplementary material

## Data Availability

The analysis code is available from the corresponding author upon reasonable request.

## Abbreviations

AU: Carea under the curve
CIs: confidence intervals
COGNISTAT: neurobehavioral cognitive status examination
DSM-5: Diagnostic and Statistical Manual of Mental Disorders, 5th edition
FDR: false discovery rate
FTD: formal thought disorder
GEE: generalized estimating equation
GiNZAa: Japanese UD-based parser
HCs: healthy controls
ICC: intraclass correlation coefficient
ICD-10: International Statistical Classification of Diseases and Related Health Problems, 10th edition
IQR: interquartile range
LSA: latent semantic analysis
NLP: natural language processing
OR: odds ratio
PANSS: Positive and Negative Syndrome Scale
PC: partial conjunction
SCZ: schizophrenia
SD: standard deviation
TF-IDF: term frequency–inverse document frequency
UD: universal dependencies
UNDERPIN: understanding psychiatric illness through natural language processing
VIF: variance inflation factor
VPTA: visual perception test for agnosia
WAB: western aphasia battery

## Supplementary material

The supplementary material for this article can be found at http://doi.org/10.1017/S0033291726104668.

## Table 1. Long description

From the top row downward, the table lists variables in the first column: Age (years), Gender (female), PANSS total, PANSS positive, PANSS negative, PANSS general psychopathology, Visits per participant (median [I QR]), Task participation for Free conversation (Task 1), Storytelling (Task 2), Picture description (Task 3), and Education (years). For each variable, values are presented for SCZ (n equals 104) and HC (n equals 101), with the third column showing p-values. Age is 48.3 plus or minus 12.7 for SCZ and 51.3 plus or minus 18.1 for H C, p equals 0.166. Gender (female) is 50 (48.1 percent) for SCZ and 56 (55.4 percent) for H C, p equals 0.360. PANSS total is 63.3 plus or minus 18.0 for SCZ and 30.7 plus or minus 1.3 for H C, p less than 0.001. PANSS positive is 15.4 plus or minus 6.0 for SCZ and 7.2 plus or minus 0.5 for H C, p less than 0.001. PANSS negative is 16.4 plus or minus 6.3 for SCZ and 7.3 plus or minus 0.4 for H C, p less than 0.001. PANSS general psychopathology is 31.4 plus or minus 8.6 for SCZ and 16.3 plus or minus 0.7 for H C, p less than 0.001. Visits per participant (median [I QR]) are 2 [1–4] for SCZ and 2 [2–2] for H C, p equals 0.096. Task participation for Free conversation (Task 1) is 104 for SCZ and 101 for HC; Storytelling (Task 2) is 101 for SCZ and 100 for HC; Picture description (Task 3) is 102 for SCZ and 101 for HC; p-values are not provided for these tasks. Education (years) is 13.3 plus or minus 2.4 for SCZ (N equals 51) and 15.4 plus or minus 2.8 for HC (N equals 70), p less than 0.001. The note at the bottom states values are mean plus or minus SD unless otherwise indicated, and education years were available for a subset.

Navigate back to Table 1..

## Table 2. Long description

Starting from the top row, Factor F1 lists Interjection ratio, Discourse ratio, and General Interjection ratio as high-loading features, with Interjection ratio as the representative feature. F2 includes Adverb ratio, Adverbial Modifier ratio, and Mean Pairwise Sentence Distance, with Adverb ratio as representative. F3 shows Normalized Tree Height, Average Sentence Length, and Normalized Dependency Distance, with Normalized Tree Height as representative. F4 presents Sentence-final Particle ratio, Particle ratio, and Marker ratio, with Sentence-final Particle ratio as representative. F5 lists Number of Sentences, Redundancy, and Vocabulary ratio, with Number of Sentences as representative. F6 includes Mean Pairwise Word Similarity, Average Distance of Semantic Graph, and Variance Pairwise Word Similarity, with Mean Pairwise Word Similarity as representative. F7 shows Case Particle ratio, Adposition ratio, and Object ratio, with Case Particle ratio as representative. F8 presents Nominal Modifier ratio, Noun ratio, and Suffix ratio, with Nominal Modifier ratio as representative. F9 lists Prenominal Adjective ratio, Filler Interjection ratio, and Determiner ratio, with Prenominal Adjective ratio as representative. All features were standardized before analysis, and factor extraction was followed by Varimax rotation. Horn’s parallel analysis indicated a nine-factor solution.

Navigate back to Table 2..

## Figure 2. Long description

From top to bottom, the y-axis lists Age, Gender (female equals one), Number of Sentences, Mean Pairwise Word Similarity, Adverb Ratio, Interjection Ratio, Case Particles (Kakujoshi) Ratio, Sentence-final Particles (Shuujoshi) Ratio, Prenominal Adjective Ratio, Nominal Modifier Ratio, and Normalized Tree Height. Each variable is represented by a horizontal line with a central open circle, indicating the odds ratio and its 95 percent confidence interval. The x-axis is labeled Odds Ratio (95 percent C I), ranging from 0.25 to 4, with a vertical dashed line at one. Most confidence intervals cross or are close to the reference line at one, except for Mean Pairwise Word Similarity, which is shifted rightward, and Case Particles (Kakujoshi) Ratio, which is shifted leftward. Gender (female equals one) and Adverb Ratio have the widest intervals. The plot visually compares the effect sizes and uncertainty for each variable.

Navigate back to Figure 2..

## Figure 3. Long description

From top to bottom, the y-axis lists: Mean Pairwise Word Similarity, Average Network Closeness Centrality, Adverb Ratio, Adverbial Modifier Ratio, Case Particle Ratio, Normalized Dependency Distance, Redundancy, Discourse Ratio, Global Perseveration, Interjection Ratio, and Average Sentence Length. The x-axis is labeled Odds Ratio (95 percent C I), spanning approximately 0.1 to 8. For each feature, three tasks are plotted: Task 1 as open circles with solid lines, Task 2 as filled diamonds with dashed lines, and Task 3 as open triangles with dotted lines. Each symbol is centered at the odds ratio estimate, with horizontal lines showing the 95 percent confidence interval. For example, Global Perseveration for Task 2 shows the largest odds ratio, extending beyond 4, while Adverb Ratio for Task 1 is below 1. Most features cluster near an odds ratio of 1, but several, such as Global Perseveration and Adverb Ratio, show wide confidence intervals. The legend at the lower right identifies the symbols for each task.

Navigate back to Figure 3..
