# Supplementary material for: An integrative NLP framework identifies multilevel linguistic phenotypes of schizophrenia across tasks
Source: Psychol Med. 2026 Jun 23;56:e193. doi: 10.1017/S0033291726104668 (PMC13319483; doi:10.1017/S0033291726104668)
Supplement: Nakamura et al. supplementary material [file S0033291726104668sup001.docx]

**Supplementary Materials**

**Table of Contents**

**S1. Supplementary Methods**

**S1-1. Speech Data Processing and Feature Extraction**

**S1-1-1. Morphological Analyses**

**S1-1-2. Syntactic Analyses**

**S1-1-2.1. Dependency Relations**

**S1-1-2.2. Syntactic Structures**

**S1-1-2.2.1. Dependency Parsing Model**

**S1-1-2.2.2. Preprocessing for Dependency Parsing**

**S1-1-2.2.3. Dependency Structure**

**S1-1-2.2.4. Center-Embedding Structure**

**S1-1-3. Semantic Analyses**

**S1-1-3.1. Word Similarity**

**S1-1-3.2. Word2Vec model details**

**S1-1-3.3. Semantic Network Analysis**

**S1-1-4. Contextual Analyses**

**S1-1-5. Overview of all NLP features and Definitions**

**S1-2. Data Partitioning and Preprocessing**

**S1-3. Statistical Analyses**

**S1-3-1. Exploratory Analyses**

**S1-3-2. Exploratory Factor Analysis (EFA)**

**S1-3-2.1. Determination of the number of factors (Horn’s PA)**

**S1-3-2.2. Factor extraction and rotation**

**S1-3-2.3. Representative-feature selection**

**S1-3-2.4. Bootstrap-based stability analysis**

**S1-3-3. Task-specific GEE Validation & Predictive Performance**

**S1-3-4. Cross-task Consistency Analyses**

**S1-4. Inclusion and Exclusion Criteria**

**S2. Supplementary Results**

**S2-1. Distributional Properties of NLP Features**

**S2-2. Exploratory Analyses**

**S2-2-1. Univariate GEE**

**S2-2-2. Correlational analysis**

**S2-3. Factor Analysis Results**

**S2-3-1. Horn’s parallel analysis results**

**S2-3-2. Factor loading matrices**

**S2-3-3. Representative feature tables**

**S2-3-4. Model Adequacy Diagnostics**

**S2-3-5. Factor stability**

**S2-3-6. Summary of Representative Features and Factor Interpretations**

**S2-4. Task-specific Validation & Predictive Performance**

**S2-5. Sensitivity Analysis: GEE Model Using Factor Scores**

**S2-6. Cross-task Robustness**

**S2-7. Multicollinearity Diagnostics**

**S1. Supplementary Methods**

**S1-1. Speech Data Processing and Feature Extraction**

**S1-1-1. Morphological analyses**

We performed Japanese morphological analyses using spaCy (version 3.4.4) (Honnibal & Montani, 2017). For written-language processing, we employed the GiNZA model ja_ginza_electra (version 5.1.2) (Megagon Labs, 2023). Morphological tokenization and part-of-speech tagging were carried out using SudachiPy (version 0.6.7) (Suzuki & Nagata, 2018) with the SudachiDict-core dictionary (version 20230110) (Works Applications, 2023). The total number of morphemes and the counts of each part-of-speech category were extracted, with the total morpheme count used as an index of speech volume.

All morphological features were normalized by utterance length, defined as the total number of morphemes. Average Sentence Length was defined as the number of morphemes per sentence, with sentence boundaries determined by human reading-mark annotations rather than GiNZA’s automatic detection, given the spoken nature of the data. Average Word Length was calculated as the number of characters per morpheme. Following previous work, Redundancy was defined as the ratio of total morphemes to unique morphemes (Toto, Murata, Tokuhisa, and Ma, 2014). We also extracted the number of named entities referring to time, place, person, and deities using GiNZA’s named-entity recognition.

**S1-1-2. Syntactic Analyses**

**S1-1-2.1. Dependency Relations**

We counted the number of dependency (modifying) relations provided by GiNZA’s dependency parser. Each feature was normalized by utterance length, defined as the total number of morphemes. For this analysis, we used the standard GiNZA model designed for written Japanese, because dependency relations are generally stable and reliably extracted even with a written-language model.

**S1-1-2.2. Syntactic Structures**

**S1-1-2.2.1. Dependency Parsing Model**

To assess syntactic complexity, we quantified two structural indices—Dependency Tree Height and Center-Embedding—using GiNZA. In these analyses, dependency parsing was performed using a Japanese conversational-speech model (ja_cejc_dropped_morph_parser-3.4.3 (version 3.4.3)) (Omura, Matsuda, Asahara, and Wakasa, 2022) based on spaCy/GiNZA, trained on the Corpus of Everyday Japanese Conversation (CEJC) (Koiso et al., 2022) developed by the National Institute for Japanese Language and Linguistics. This model is optimized for spoken Japanese and provides robust parsing of sentence-final particles and elliptical constructions that frequently occur in natural conversation.

S1-1-2.2.2. Preprocessing for Dependency Parsing

Before parsing, tokens tagged as “interjection-filler” or assigned the part-of-speech “INTJ” were removed, and Japanese punctuation marks (“、”, “。”) were deleted. Sentences containing fewer than five tokens were excluded from analysis.

**S1-1-2.2.2. Dependency Structure**

Dependency relations identified by GiNZA were counted. In the dependency structure, each token (morpheme) was treated as a “node,” and dependency links were represented as “edges.” Nodes without incoming edges were defined as “roots,” and those without outgoing edges as “leaves.” Tree height was defined as the maximum distance from a leaf to its root, and both the maximum and total tree heights per utterance were calculated from these structures. Additionally, we calculated the dependency distance, which is the absolute positional difference between a dependent and its head within a sentence. The total dependency distance per utterance was used to measure linear syntactic complexity.

**S 1-1-2.2.3. Center-Embedding Structure**

We computed two indices quantifying hierarchical syntactic complexity in patient speech based on dependency parsing: Maximum Center-Embedding Depth and Center-Embedding Count. Each analysis unit corresponded to one subject, one visit, and one task.

*Definition of pure containment*

Each dependency relation was represented as an edge ‘(s,t)’ (token index, head index), excluding self-references. Each edge was normalized into an interval ‘(L, R)’ where ‘L = min(s,t)’ and ‘R = max(s,t)’. Degenerate and duplicate edges were removed. “Pure containment” was defined such that ‘(L1,R1)’ strictly contained ‘(L2,R2)’ when ‘L1 < L2 < R2 < R1’. Overlaps and shared endpoints were excluded. Containment was recognized only when both edges had the same direction (rightward or leftward).

*Maximum Center-Embedding Depth*

All dependency edges within a sentence were treated as nodes, and pure-containment relations as directed edges, forming a hierarchical containment graph. Starting from each edge, we recursively traversed contained edges to obtain the most extended reachable chain length. The maximum chain length per sentence was defined as its Center-Embedding Depth. The highest such value across all sentences in the same file (ID–Visit–Part) was recorded as Maximum Center-Embedding Depth, representing the greatest degree of hierarchical syntactic embedding in the utterance.

Center-Embedding Count

For each sentence, the maximum nesting depth was computed, and these values were summed across all sentences within the file to yield the Center-Embedding Count. Thus, Maximum Center-Embedding Depth captures peak syntactic complexity, while Center-Embedding Count reflects the overall frequency and accumulation of nested structures.

**S 1-1-3. Semantic analyses**

**S 1-1-3.1. Word Similarity**

Word2Vec embeddings (Mikolov, Chen, Corrado, and Dean, 2013) were used to assess variability and bias in lexical usage among participants with schizophrenia. Texts were first segmented into morphemes using MeCab (Kudo, Yamamoto, and Matsumoto, 2004) with the mecab-ipadic-NEologd (Sato, Hashimoto, and Okumura, 2017) dictionary, retaining only content words. Unique content words were then vectorized using a Word2Vec model trained on the 2017 Japanese Wikipedia corpus (Suzuki, 2018). For each task, cosine similarities between content-word vectors were calculated in two ways: (1) between adjacent content words and (2) across all pairwise combinations. Additionally, we measured the repeated use of content words in two ways. Adjacent Duplicates refers to the number of identical content-word pairs that appear consecutively within an utterance. Conversely, Global Duplicates counts the total number of identical content-word pairs across the utterance.

**S 1-1-3.2. Word2Vec Model Details**

We used the Japanese Wikipedia Entity Vector model (Suzuki, 2018), a pre-trained Word2Vec embedding trained on the full text of the Japanese Wikipedia. Wikipedia articles were extracted using WikiExtractor, tokenized with MeCab, and trained using the skip-gram with negative sampling (SGNS) algorithm implemented in Word2Vec [8].

Both words and Wikipedia entities were represented in a shared 200-dimensional continuous vector space. The training parameters were set as follows: vector size = 200, context window = 5, subsampling threshold = 1 × 10⁻³, negative samples = 5, and hierarchical softmax disabled. This publicly available model provides distributed representations of Japanese words and entities, trained on the entire Japanese Wikipedia corpus. Given our focus on relative semantic distances within a single embedding space and on group-level comparisons, we selected a well-established Japanese Word2Vec model and did not perform cross-architecture comparisons.

**S 1-1-3.3. Semantic Network Analysis**

Using the comprehensive UNDERPIN dataset (Kishimoto et al., 2022), we constructed separate Term Frequency–Inverse Document Frequency (TF-IDF) models (Salton & Buckley, 1988) for each task.

Each participant’s speech sample from a single visit and task was treated as a single document, and the content words within it were used as tokens for model training.

Then, we extracted the top 10% high-score (TF-IDF score) words from the subject’s speech. According to the previous study (Sasahara, 2016), we formed graphs with content words as nodes, setting the top 10% high-scoring words as the initial nodes; we connected *N=10* words exceeding the sth = 0.3 similarity threshold until fewer than N=10 remained (using NetworkX in Python for graph creation). This threshold was chosen because the mean word similarity across the entire UNDERPIN corpus was 0.28, ensuring that the network captured semantically meaningful associations.

Finally, we calculated the following indicators using network analysis methods: the number of nodes, the ratio of words selected for the nodes, the clustering coefficient, the density, the diameter, the average distance, and the average closeness centrality.

**S 1-1-4. Contextual analyses**

Sentence-BERT is a sentence embedding model that utilizes a pre-trained BERT with a Siamese network architecture. The model employs mean pooling of token embeddings from a BERT backbone to generate 768-dimensional sentence representations. For this study, we used the Japanese Sentence-BERT model (Sonoisa, 2020), a Japanese adaptation of Sentence-BERT trained on large-scale Japanese corpora and implemented using the Hugging Face Transformers library. The textual parts of each participant’s speech in each task were segmented into sentences, and each sentence was vectorized using this model. Subsequently, cosine distances were calculated in two ways: (1) between adjacent sentences and (2) across all pairwise combinations. In both analyses, cosine distances below 0.05 were considered perseveration.

**S1-1-5. Overview of all NLP features and Definitions**

All 76 NLP features and their definitions are shown in Table S1.

| **analysis** | **features** | **feature description** |
| --- | --- | --- |
| Morphological analysis | Number of Morphemes | Total number of morphemes in the text |
|  | Number of Sentences | Total number of sentences in the text |
|  | Noun Ratio | Ratio of nouns among all morphemes |
|  | Proper Noun Ratio | Ratio of proper nouns among all morphemes |
|  | Verb Ratio | Ratio of verbs among all morphemes |
|  | Adjective Ratio | Ratio of adjectives among all morphemes |
|  | Adverb Ratio | Ratio of adverbs among all morphemes |
|  | Interjection Ratio | Ratio of interjections among all morphemes |
|  | Pronoun Ratio | Ratio of pronouns among all morphemes |
|  | Numeral Ratio | Ratio of numerals among all morphemes |
|  | Auxiliary Verb Ratio | Ratio of auxiliary verbs among all morphemes |
|  | Coordinating Conjunction Ratio | Ratio of coordinating conjunctions among all morphemes |
|  | Subordinating Conjunction Ratio | Ratio of subordinating conjunctions among all morphemes |
|  | Determiner Ratio | Ratio of determiners among all morphemes |
|  | Adposition Ratio | Ratio of adpositions among all morphemes |
|  | Particle Ratio | Ratio of particles among all morphemes |
|  | Punctuation Ratio | Ratio of punctuation marks among all morphemes |
|  | Symbol Ratio | Ratio of symbols among all morphemes |
|  | General Interjection Ratio | Ratio of general interjections among all morphemes |
|  | Filler Interjection Ratio | Ratio of filler-type interjections among all morphemes |
|  | Case Particle (Kakujoshi) Ratio | Ratio of case particles (kakujoshi) among all morphemes |
|  | Adverbial Particle (Fukujoshi) Ratio | Ratio of adverbial particles (fukujoshi) among all morphemes |
|  | Focus Particle (Kakarijoshi) Ratio | Ratio of focus particles (kakarijoshi) among all morphemes |
|  | Conjunctive Particle (Setuzokujoshi) Ratio | Ratio of conjunctive particles (setuzokujoshi) among all morphemes |
|  | Sentence-Final Particle (Shuujoshi) Ratio | Ratio of sentence-final particles (shuujoshi) among all morphemes |
|  | Prenominal Adjective (Rentaishi) Ratio | Ratio of prenominal adjectives (rentaishi) among all morphemes |
|  | Prefix (Settouji) Ratio | Ratio of prefixes (settouji) among all morphemes |
|  | Suffix (Setsubiji) Ratio | Ratio of suffixes (setsubiji) among all morphemes |
|  | Average Sentence Length | Mean number of morphemes per sentence |
|  | Average Word Length | Mean number of characters per morpheme |
|  | Redundancy | Ratio of total morpheme count to the number of unique morpheme types |
| Contextual features | Mean Adjacent Sentence Cosine Distance | Mean cosine distance between adjacent sentences |
|  | Variance of Adjacent Sentence Cosine Distance | Variance of cosine distances between adjacent sentences |
|  | Adjacent Perseveration ratio | Ratio of adjacent sentence pairs with cosine distance < 0.05 |
|  | Mean Pairwise Sentence Distance | Mean cosine distance across all pairwise combinations of sentences |
|  | Variance of Pairwise Sentence Distance | Variance of cosine distances across all pairwise combinations of sentences |
|  | Global Perseveration ratio | Ratio of all sentence pairs with cosine distance < 0.05 |

| **analysis** | **features** | **feature description** |
| --- | --- | --- |
| Syntactic analysis | Maximum Center‑Embedding Depth | Maximum nesting depth (center‑embedding) observed in the dependency structure |
|  | Maximum Dependency Tree Height | Maximum height of the dependency tree across sentences |
|  | Normalized Center‑Embedding Count | Total number of center‑embedding instances divided by the morpheme count |
|  | Normalized Dependency Distance | Sum of dependency distances divided by the morpheme count |
|  | Normalized Tree Height | Sum of dependency tree heights divided by the morpheme count |
|  | Clausal Modifier of Noun Ratio | Ratio of tokens labeled as clausal modifiers of nouns (UD label: acl) |
|  | Adverbial Clause Modifier Ratio | Ratio of tokens labeled as adverbial clause modifiers (UD label: advcl) |
|  | Adverbial Modifier Ratio | Ratio of tokens labeled as adverbial modifiers (UD label: advmod) |
|  | Adjectival Modifier Ratio | Ratio of tokens labeled as adjectival modifiers (UD label: amod) |
|  | Case Marking Ratio | Ratio of tokens labeled as case markers (UD label: case) |
|  | Coordinating Conjunction Ratio (Syntax) | Ratio of tokens labeled as coordinating conjunctions (UD label: cc) |
|  | Clausal Complement Ratio | Ratio of tokens labeled as clausal complements (UD label: ccomp) |
|  | Compound Ratio | Ratio of tokens labeled as compounds (UD label: compound) |
|  | Clausal Subject Ratio | Ratio of tokens labeled as clausal subjects (UD label: csubj) |
|  | Determiner Ratio (Syntax) | Ratio of tokens labeled as determiners (UD label: det) |
|  | Discourse Element Ratio | Ratio of tokens labeled as discourse elements (UD label: discourse) |
|  | Marker Ratio | Ratio of tokens labeled as markers (UD label: mark) |
|  | Nominal Modifier Ratio | Ratio of tokens labeled as nominal modifiers (UD label: nmod) |
|  | Nominal Subject Ratio | Ratio of tokens labeled as nominal subjects (UD label: nsubj) |
|  | Numeric Modifier Ratio | Ratio of tokens labeled as numeric modifiers (UD label: nummod) |
|  | Object Ratio | Ratio of tokens labeled as objects (UD label: obj) |
|  | Oblique Nominal Ratio | Ratio of tokens labeled as oblique nominals (UD label: obl) |
|  | Overridden Disfluency Ratio | Ratio of tokens labeled as overridden disfluencies (UD label: reparandum) |
| Named Entity Recognition | Named Entity (DEITY) Ratio | Ratio of tokens identified as named entities of type DEITY |
|  | Named Entity (LOCATION) Ratio | Ratio of tokens identified as named entities of type LOCATION |
|  | Named Entity (TIME/DATE) Ratio | Ratio of tokens identified as named entities of type TIME/DATE |
|  | Named Entity (PERSON) Ratio | Ratio of tokens identified as named entities of type PERSON |
| Word Similarity using word2Vec embeddings | Mean Adjacent Word Similarity | Mean adjacent word similarity |
|  | Variance of Adjacent Word Similarity | Variance of adjacent word similarity |
|  | Adjacent Duplicates ratio | Ratio of adjacent identical content word pairs |
|  | Global Duplicates ratio | Ratio of all identical content word pairs |
|  | Mean Pairwise Word Similarity | Mean pairwise word similarity |
|  | Variance of Pairwise Word Similarity | Variance of word similarity |
|  | Vocabulary ratio | Ratio of unique content words |
| Network Analysis of Semantic Graph | Clustering Coefficient of Semantic Graph | Cluster coefficient of a semantic graph |
|  | Average Closeness Centrality of Semantic Graph | Average of closeness centrality of a semantic graph |
|  | Average Distance of Semantic Graph | Average of distance of a semantic graph |
|  | Diameter of Semantic Graph | Diameter of a semantic graph |
|  | Density of of Semantic Graph | Density of a semantic graph |

Table S1. Overview of all NLP features and Definitions

**S1-2. Data Partitioning and Preprocessing**

*Preprocessing of linguistic features*

Among the 76 linguistic features initially extracted, those whose values were zero for all samples were excluded due to the lack of informational value. When necessary, features were normalized by the Number of Morphemes to account for differences in utterance length. Missing values were infrequent; when present, they were imputed using the global mean of each feature.

*Statistical Characterization of Linguistic Features*

To summarize the hierarchical structure and statistical characteristics of all linguistic features, we calculated descriptive and reliability metrics separately for each part of the dataset. For each numeric feature, we measured (i) the hierarchical structure resulting from repeated visits nested within participant IDs by estimating the between-subject and within-subject variance components and intraclass correlation coefficients (ICC) using a random-intercept mixed-effects model (MixedLM); (ii) test–retest correlations between the first two visits with Spearman’s ρ; (iii) distributional traits such as mean, SD, median, IQR, skewness, and kurtosis; (iv) normality using Shapiro–Wilk or D’Agostino’s K² test depending on sample size; (v) variance homogeneity between diagnostic groups with Levene’s test; and (vi) outlier rates using a median absolute deviation (MAD > 3) criterion. Missing data was also assessed for each feature. All findings were organized into part-specific summary tables and exported as Excel files.

*ID-level partitioning and construction of fixed train/test splits.*

To ensure rigorous evaluation of model generalizability and to avoid subject-level data leakage, the dataset was partitioned into training and test sets at the participant ID level. All observations for the same participant (including multiple visits) were assigned to the same partition. The partitioning was performed separately for each Part defined in the dataset, with an 80%/20% split into training and test sets. The resulting assignments were stored as fixed partition columns (e.g., partition_P1, partition_P2) and used consistently throughout all subsequent analyses.

*Balancing demographic and categorical distributions*

The partitioning procedure was optimized to minimize distributional imbalances between the training and test sets. Age was converted to a numeric variable, and the algorithm evaluated between-set differences in (i) the mean, (ii) the standard deviation, and (iii) an approximate p-value from a two-sample Kolmogorov–Smirnov test, yielding a composite imbalance score for age. For categorical variables—site, gender, and diagnosis—the algorithm computed L1 distances between the training and test distributions at both the participant level (unique IDs) and the observation level (rows). When an explicit site variable was unavailable, the site was automatically inferred from the second character of the participant ID, following the structure of the dataset. Visit distributions were also compared between sets using the L1 distance to encourage comparable visit proportions across partitions.

*Multi-trial optimization of partition assignments*

For each Part, the algorithm repeatedly generated random ID-level assignments, allocating approximately 20% of participants to the test set. This random assignment was repeated 2,048 times using a fixed global random seed to ensure reproducibility. For every trial, the imbalance score—incorporating age, categorical variables, and visit distribution—was calculated, and the assignment with the lowest overall imbalance score was selected as the final partition.

*Downstream analysis*

All feature selection and model estimation procedures were conducted exclusively within the training set, using the fixed partitions described above. The test set was used solely to evaluate out-of-sample model performance.

**S1-3. Statistical Analyses**

**S1-3-1. Exploratory Analyses**

*Exploratory Group Comparison*

Group differences in each linguistic feature were tested using univariate GEE models with diagnosis (SCZ vs. HC) as the predictor. Age and gender were included as covariates, and participant ID was used as the clustering variable. Odds ratios (ORs) and corresponding p-values were obtained, and multiple-comparison correction was performed using the Benjamini–Hochberg FDR. Features with FDR < 0.05 were considered significant.

*Correlation Analysis with PANSS Domains*

Spearman correlations were computed between all linguistic features and five PANSS domains. Correlation coefficients and p-values were obtained, followed by FDR correction across all tests. Features meeting FDR < 0.05 and |r| ≥ 0.20 were retained for further interpretation.

**S 1-3-2. Exploratory Factor Analysis (EFA)**

Analyses used the training set. For each participant, feature values were averaged across available recordings to form a subject-by-feature matrix. Zero-variance features after aggregation were removed. Columns were z standardized. We computed the empirical Pearson correlation matrix (R) (diagonal forced to 1.0) for diagnostics and for inter-representative redundancy checks.

**S1-3-2.1. Determination of the number of factors (Horn’s PA)**

The number of factors was determined using Horn’s correlation-based parallel analysis. Eigenvalues from the observed standardized covariance matrix were compared with the 99th-percentile eigenvalues generated from 2,000 random normal datasets matched in dimension. Factors with observed eigenvalues exceeding their null thresholds were retained. Random seeds were fixed.

**S1-3-2.2. Factor extraction and rotation**

Factors were estimated using scikit-learn’s *FactorAnalysis* with EM optimization (fixed random seed). The loading matrix was orthogonally rotated using a Varimax transformation. Model adequacy in the standardized space was assessed by comparing the observed correlation matrix with the reproduced matrix R^=ΛvarimaxΛvarimaxT+diag(ψ), examining residual correlations and their norms.

**S1-3-2.3. Representative-feature selection**

To avoid factor-score indeterminacy and enhance interpretability, one representative feature was selected per factor. A feature is qualified if it meets all of the following criteria: (i) a primary absolute loading ≥ 0.60; (ii) a loading gap ≥ 0.20 relative to the second-highest loading; and (iii) low redundancy with previously selected representatives (|r| < 0.80).
If no feature satisfied all criteria, thresholds were relaxed in a predefined sequence: first the loading-gap requirement, then the loading magnitude, and finally the redundancy threshold (allowing |r| ≤ 0.95). If multiple candidates remained, the feature with the highest primary loading was selected.
For each factor, we recorded the selected representative, its loadings, and any relaxation applied during selection.

**S1-3-2.5. Stability Assessment via Resampling**

Before evaluating stability, overall model adequacy was examined by comparing the observed correlation matrix with the reproduced matrix from the Varimax-rotated factor model, assessing both the maximum absolute residual correlation and the Frobenius norm of the residual matrix.

Factor-solution stability was then evaluated by repeating the full pipeline (parallel analysis → factor extraction → Varimax rotation → representative-feature selection) under two resampling schemes: (i) bootstrap resampling of subjects (2,000 iterations), and (ii) 70% Monte-Carlo subsampling (2,000 iterations). Because factor solutions are rotationally indeterminate, each resampled loading matrix was Procrustes-aligned to the full-sample rotated solution. Stability was summarized using Tucker’s factor congruence and representative-feature selection frequencies. All results, including aligned loadings, residual-fit diagnostics, and summary statistics, were archived with analysis parameters for reproducibility.

**S1-3-3. Task-specific GEE-validation & Predictive performance**

For Task 1, we estimated a task-specific GEE classifier using the representative linguistic features derived from the EFA pipeline. Age and gender were included as covariates, and participant ID was used as the clustering variable. The model was fitted to the Task 1 training set using a binomial logit GEE with a robust, independent working correlation. Predicted probabilities were obtained for the corresponding held-out test set to evaluate discrimination (record-level and patient-level AUC) and threshold-based classification metrics. The optimal patient-level threshold was determined in the training set using Youden’s J statistic and then applied to the test set. Significance of the patient-level AUC was assessed by a label-permutation test with 5,000 repetitions, and 95% percentile confidence intervals were obtained by subject-level cluster bootstrapping with 2,000 resamples. The same cluster bootstrap scheme (2,000 resamples) was also used to derive percentile confidence intervals for sensitivity, specificity, positive and negative predictive values, accuracy, F1 score, and balanced accuracy at the fixed threshold.

To assess robustness, the same procedure was repeated using factor scores (from the task-specific EFA solution) instead of the representative features. Coefficients and OR were compared to evaluate sensitivity to feature representation. This entire sequence of analyses—comprising train-set estimation, test-set prediction, threshold determination, permutation testing, and bootstrap evaluation—was performed identically across all tasks.

**S1-3-4. Cross-task consistency analyses**

*Partial-Conjunction analysis*

Task-specific p-values from the logistic GEE models were combined across the three tasks using the partial-conjunction (PC) framework (Benjamini & Heller, 2008), which tests whether a feature shows a significant group effect in at least two tasks. PC p-values were then corrected for multiple comparisons using the Benjamini–Hochberg FDR procedure, with significance defined as q_PC_ < 0.05.

*Task-by-feature sensitivity analysis*

To test whether the diagnostic effect of each linguistic feature varied across tasks, logistic GEE models were fitted with diagnosis as the outcome and task-by-feature interaction terms as predictors, adjusting for age and gender. Features were z-standardized within each task, and participant ID was set as the clustering unit with an independent working correlation. Interaction effects were evaluated using Wald chi-square tests, with contrasts estimated via linear combinations where appropriate.

**S 1-4. Inclusion and Exclusion Criteria**

**Patients**

**Inclusion criteria**

Patients were eligible if they:

1. Met DSM-5 or ICD-10 criteria for schizophrenia.
2. Were receiving inpatient or outpatient treatment at Keio University Hospital or affiliated institutions.
3. Were aged ≥20 years at the time of consent.
4. Were judged capable of providing written informed consent by their treating psychiatrist.

**Exclusion criteria**

Patients were excluded if:

1. Participation was judged by the treating psychiatrist to pose substantial psychological or physical burden likely to affect clinical stability.
2. They had comorbid medical or neurological conditions that could interfere with speech recording (e.g., severe speech impairment or vocal cord removal).
3. The principal investigator or treating physician deemed participation inappropriate for clinical or methodological reasons.

**Healthy Controls**

**Inclusion criteria**

Healthy controls:

1. Had no history of psychiatric disorders.
2. Were aged ≥20 years.
3. Volunteered in response to recruitment advertisements.

**Exclusion criteria**

Healthy controls were excluded if they had medical or neurological conditions that could interfere with speech production.

**S2. Supplementary Results**

**S2-1. Distributional Properties of NLP Features**

We summarized the basic distributional metrics of all NLP features for each task to document their reliability, clustering structure, and data quality. One feature in Task 3 (Named Entity (DEITY) Ratio) had zero variance and was excluded from subsequent analyses.

| Task | N subjects | N observations | N Features | ICC | Design effect | Outlier rate % | Missing rate % |
| --- | --- | --- | --- | --- | --- | --- | --- |
| Task 1 | 205 | 447 | 76 | 0.60 | 1.70 | 6.70 | 0.22 |
| Task 2 | 203 | 446 | 76 | 0.36 | 1.42 | 8.07 | 0.00 |
| Task 3 | 201 | 445 | 75 | 0.47 | 1.57 | 5.84 | 0.00 |

Table S2. Distributional Properties of NLP Features

**S2-2. Exploratory Analyses**

**S2-2-1. Univariate GEE**

Univariate GEE models were fitted for all Task 1 features to explore task-specific group differences. The results are summarized in Figure S1 (volcano plot), Figure S2 (forest plot), and Table S3. Figure S3 displays raincloud plots for features that remained significant after FDR correction (q < 0.05).

**

Figure S1. Volcano plot of univariate GEE analyses on Task 1

This volcano plot shows the results of univariate generalized estimating equation (GEE) analyses examining the association between each linguistic feature in Task 1 (free conversation) and SCZ diagnosis. The x-axis displays the log₂ odds ratio (OR), while the y-axis shows the –log₁₀ p-value. Each dot represents a specific linguistic feature. Gray dots indicate features that do not reach statistical significance. Blue and red dots show features that pass false discovery rate (FDR) correction (q < 0.05), with blue representing positive and red representing negative log₂OR. Key top-ranked features are labeled. The horizontal dashed red line indicates the threshold of p = 0.05.
Footnote:

num_person_ratio = Named Entity (PERSON) Ratio.

advmod_ratio = Adverbial Modifiers Ratio.

num_adv_ratio = Adverbs Ratio.

num_adp_ratio = Adpositions Ratio.

num_kakarijoshi_ratio = Focus Particle (Kakarijoshi) Ratio.

reparandum_ratio = Overridden Disfluency Ratio.

w2vsim_mean = Mean Pairwise Word Similarity.

compound_ratio = Compound Ratio.

duplicates_adj_ratio = Adjacent Duplicates Ratio.


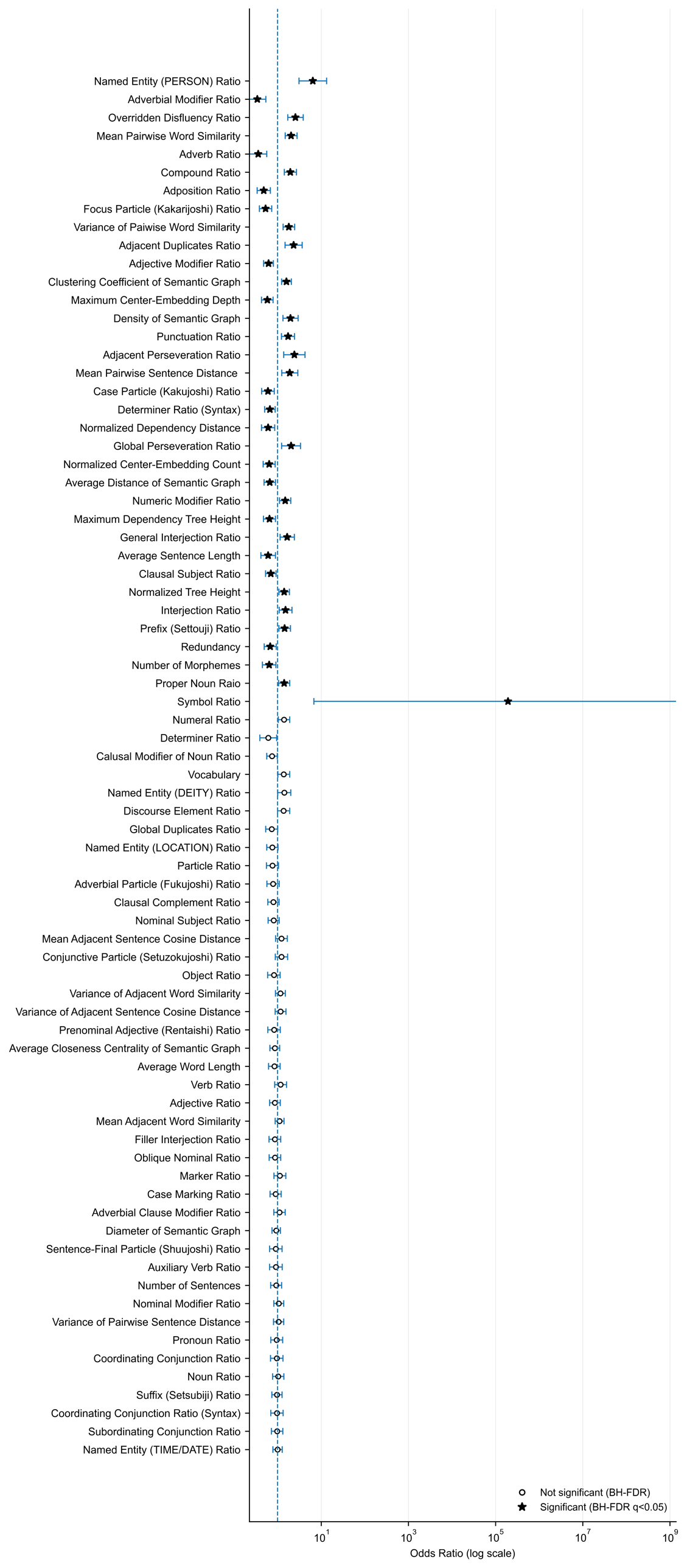


**Supplementary Figure S2. Forest plot of univariate GEE associations between linguistic features and schizophrenia diagnosis.**
Odds ratios (ORs) and 95% confidence intervals (CIs) are shown for each of the 76 features from univariate generalized estimating equation (GEE) models with a logit link, adjusting for age and sex, using an independent working correlation structure and participant-clustered robust standard errors. Features are ordered by Benjamini–Hochberg FDR–adjusted p-values (q-values) in ascending order. The vertical dashed line indicates OR = 1. Filled stars denote features significant after BH-FDR correction (q < 0.05), and open circles denote non-significant features. ORs are plotted on a logarithmic scale.

| Feature | OR (per 1 SD) | 95% CI | q-value (FDR) |
| --- | --- | --- | --- |
| Named Entity (PERSON) Ratio | 6.44 | 3.10–13.40 | < 0.001 |
| Adverb ratio | 0.36 | 0.23–0.57 | < 0.001 |
| Adverbial Modifier ratio | 0.34 | 0.22–0.54 | < 0.001 |
| Compound ratio | 1.97 | 1.42–2.72 | < 0.001 |
| Mean Pairwise Word Similarity | 2.05 | 1.49–2.80 | < 0.001 |
| Clustering Coefficient of a Semantic Graph | 1.6 | 1.24–2.07 | < 0.001 |
| Mean Pairwise Sentence Distance | 1.91 | 1.25–2.92 | 0.011 |
| Maximum Dependency Tree Height | 0.65 | 0.48–0.89 | 0.022 |

Table S3. Examples of representative linguistic features showing significant group differences in Task 1

Values are presented as odds ratios (OR) per 1 SD increase in the feature, along with 95% confidence intervals. P-values were adjusted for multiple comparisons using the false discovery rate (FDR) and reported as q-values. Features with q < 0.05 were considered statistically significant.

**
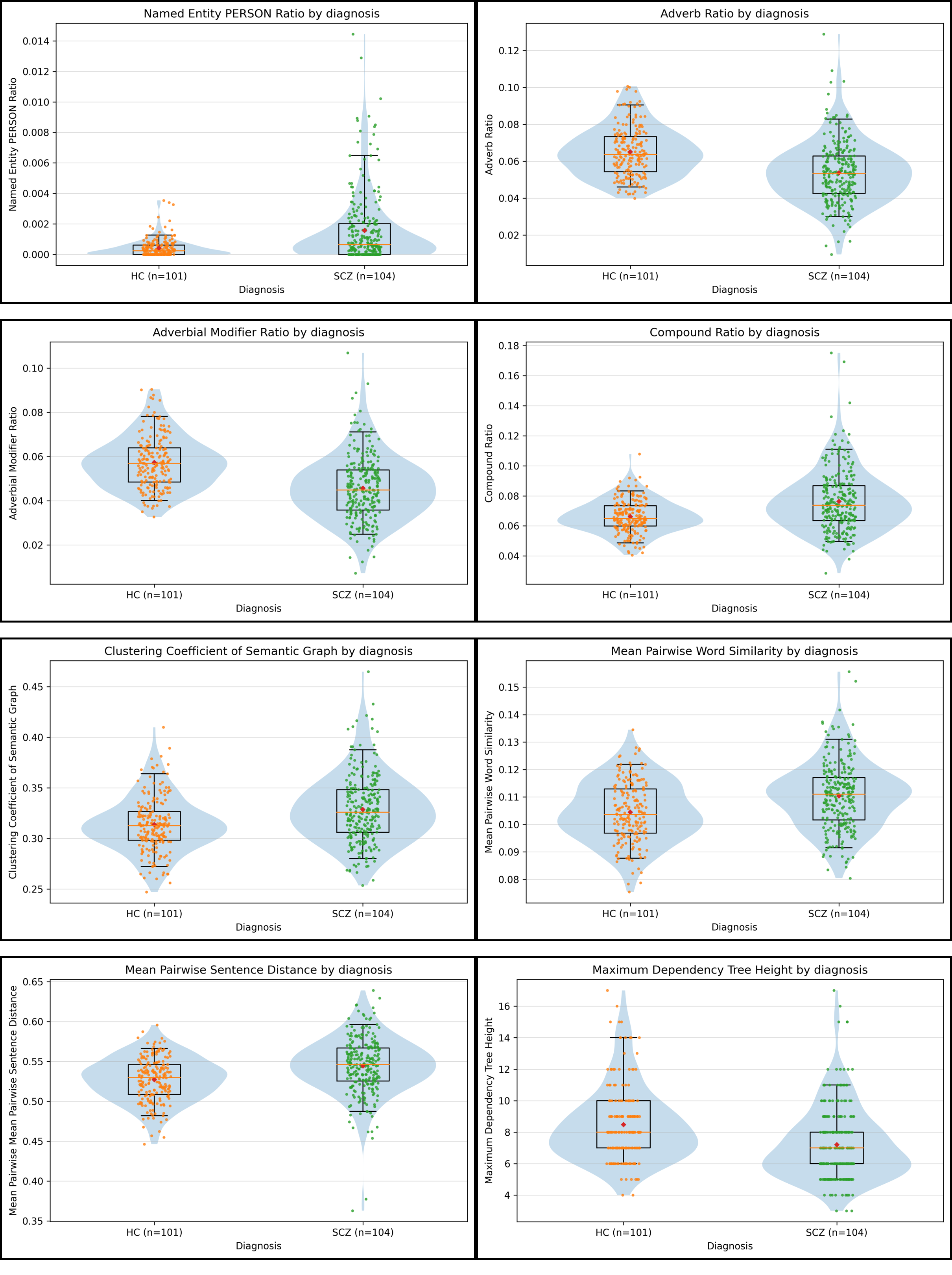
**

Figure S3. Raincloud plots of major linguistic features showing significant group differences in Task 1.

**S2-2-2. Correlational Analysis**

Figure S4 presents a subset of the exploratory correlations between linguistic features and PANSS domains in Task 1.


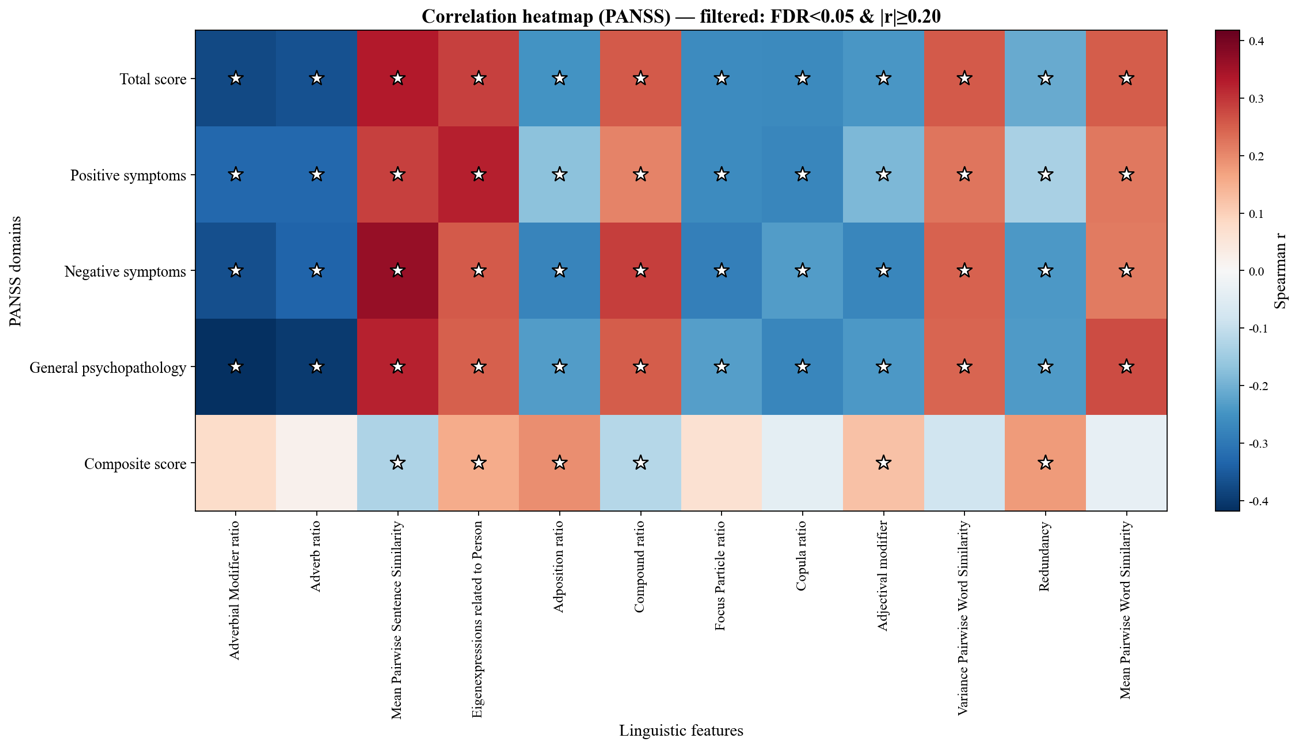


Figure S4. Exploratory correlations between linguistic features and PANSS domains in Task 1

Heatmap showing Spearman’s correlation coefficients (r) between selected linguistic features and PANSS domains (Total, Positive, Negative, and General Psychopathology). Features were included only if they demonstrated significant correlations (FDR-corrected p < 0.05, |r| ≥ 0.20) with at least three PANSS domains, thereby improving clarity and emphasizing strong associations. The color scale indicates the strength and direction of the correlation, and white stars mark FDR-significant correlations.

We examined the correlations between PANSS scores, and the three core linguistic markers identified in Task 1 (free conversation): Case Particle (Kakujoshi) Ratio, Adverb Ratio, and Mean Pairwise Word Similarity. Scatter plots display observations from each participant at each visit (repeated measures), with ordinary least squares fit lines. Panel annotations report Pearson’s r and two-sided p-values (raw p), together with the number of observations.

**Supplementary Figure S5. Correlations between core linguistic markers from Task 1 (free conversation) and PANSS scores.**
Scatter plots depict the associations between each of the three core linguistic markers identified in **Task 1 (free conversation)**—case particle ratio (Kakujoshi), mean pairwise word similarity, and adverb ratio—and PANSS scores. Columns correspond to PANSS Positive, Negative, and Total scores; rows correspond to the three markers. Each point represents an individual participant. Solid lines indicate ordinary least squares regression fits. Pearson’s correlation coefficient (r), two-sided p-values, and sample size (n) are shown in each panel.

PANSS, Positive and Negative Syndrome Scale.

**S2-3. Factor Analysis Results**

**S2-3-1. Horn’s parallel analysis results**

Horn’s parallel analysis based on the correlation matrix was conducted to determine the optimal number of factors. Observed eigenvalues of the empirical data were compared against the 99th percentile of eigenvalues from 2,000 null datasets generated from random normal variables of identical dimensions. The results are shown in Figure S6.


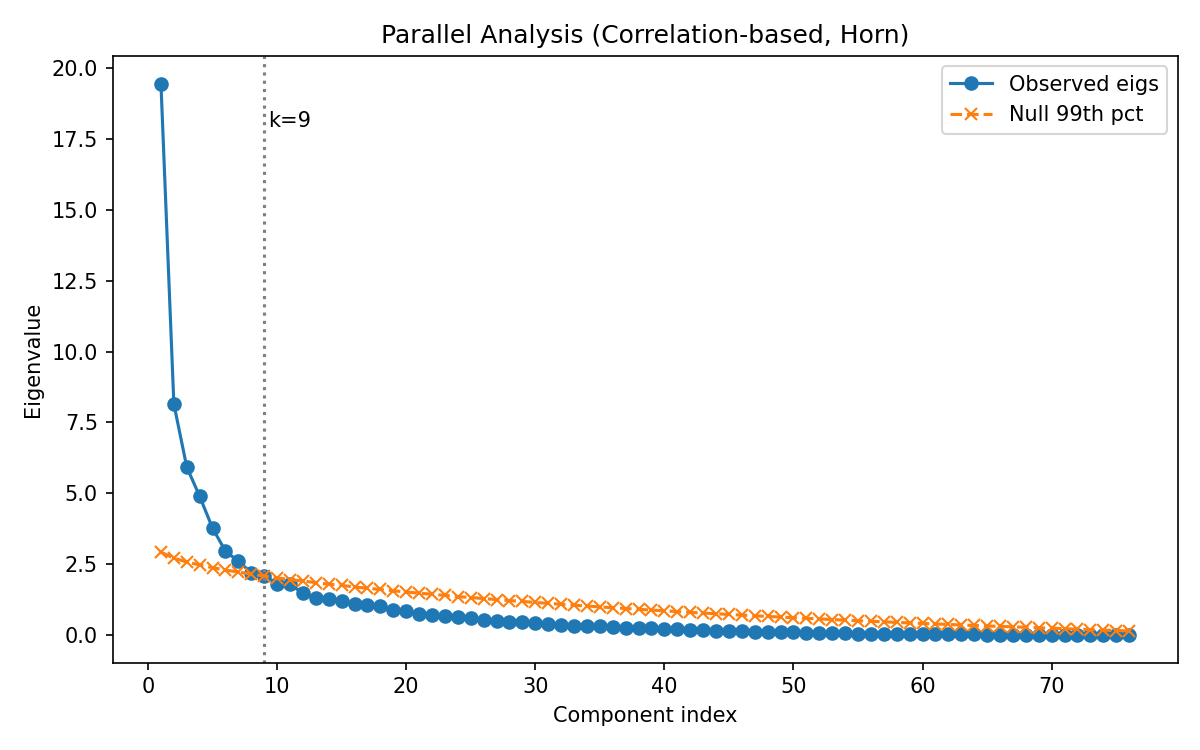

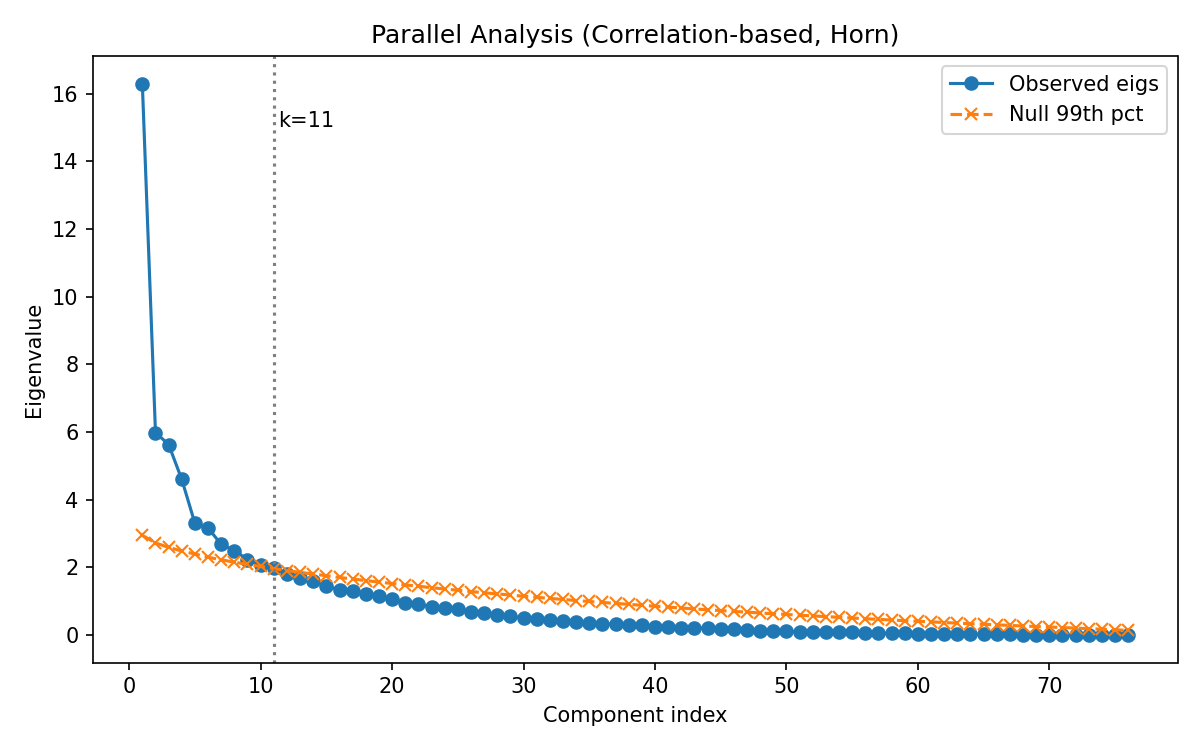

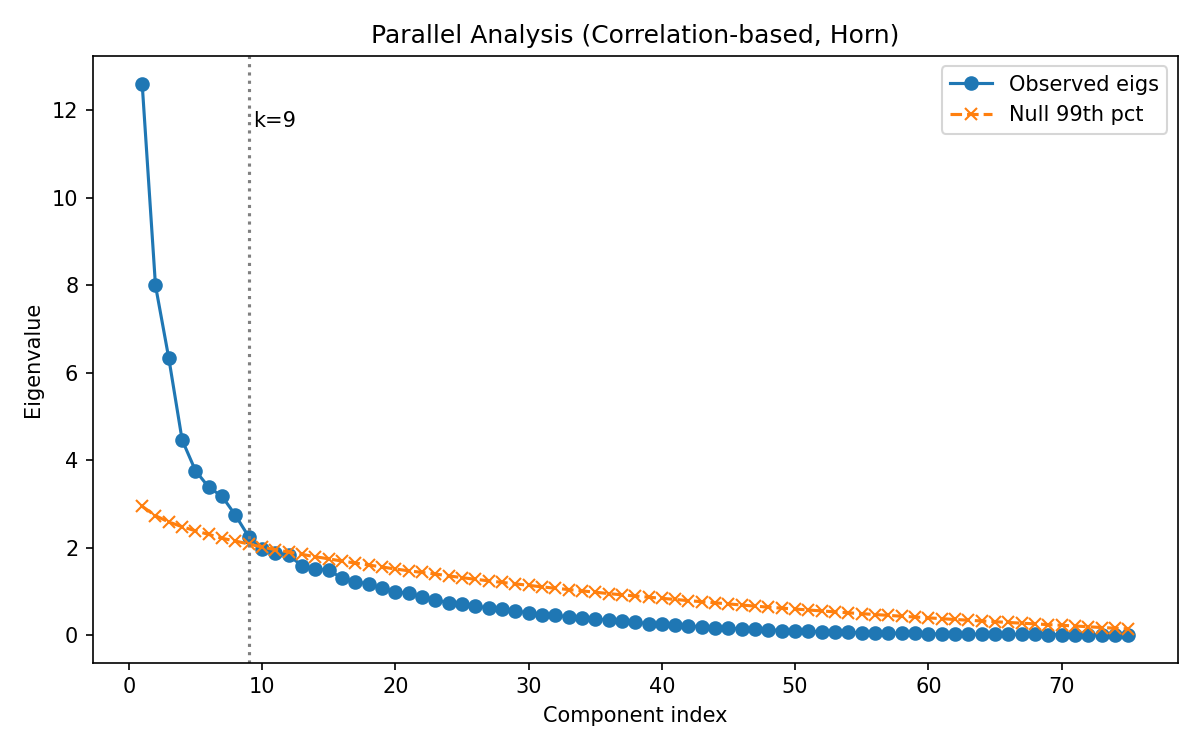


Task 1

Task 2

Task 3

Figure S6—Horn’s parallel analysis (correlation-based).

Blue circles indicate the observed eigenvalues from each task’s empirical correlation matrix, and orange crosses indicate the 99th-percentile eigenvalues derived from 2,000 Monte-Carlo null datasets. The vertical dashed line marks the point at which the observed eigenvalues fall below the null threshold. Parallel analysis yielded k = 9, 11, and 9 retained factors for Task 1, Task 2, and Task 3, respectively.

*Factor Decomposition for Tasks 2 and 3*

Task 2

The exploratory factor analysis of Task 2 indicated an 11-factor solution based on Horn’s parallel analysis. Factor 1 accounted for 7.1% of the variance, followed by Factors 2–11, each explaining between 3.4% and 7.7%. The cumulative variance explained by the 11 factors reached 58.3%, indicating a moderately distributed latent structure.

Task 3

The factor analysis of Task 3 yielded a 9-factor solution, with the first factor accounting for 3.3% of the total variance. Factors 2–9 explained an additional 4.0–11.9% of the variance each, with a cumulative explained variance of 56.7%.

**S2-3-2. Factor loading matrices (varimax)**

Tables S4–S6 present the varimax-rotated factor loading matrices for Tasks 1–3. For each factor, the features with the largest absolute loadings are summarized together with the assigned factor label. Factor labels were determined by examining the signs and magnitudes of the dominant loadings, thereby allowing interpretation of the latent linguistic construct represented by each factor.

Task 1

| **Factor1_feature** | **loadings** | **Factor2_feature** | **loadings** |
| --- | --- | --- | --- |
| Interjection Ratio | 0.93 | Adverb Ratio | 0.95 |
| Discourse Element Ratio | 0.93 | Adverb Modifier Ratio | 0.93 |
| General Interjection Ratio | 0.90 | Mean Pairwise Sentence Distance | -0.51 |
| Subordinating Conjunction Ratio | -0.83 | Mean Adjacent Sentence Distance | -0.42 |
| Punctuation Ratio | 0.82 | Noun Ratio | -0.41 |
| Adverbial Clause Modifier Ratio | -0.79 | Compound Ratio | -0.35 |
| Conjunctive Particle (Setuzokujoshi) Ratio | -0.76 | Prefix (Settouji) Ratio | -0.31 |
| Verb Ratio | 0.75 | Adjacent Perseveration Ratio | -0.30 |
| Global Perseveration Ratio | 0.74 | Verb Ratio | -0.28 |
| Global Duplicates Ratio | -0.73 | Global Perseveration Ratio | -0.27 |
| **Factor 1: Discourse factor** | | **Factor 2: Modification-Coherence factor** | |

| **Factor3_feature** | **loadings** | **Factor4_feature** | **loadings** |
| --- | --- | --- | --- |
| Normalized Tree Height | 0.85 | Sentence-Final Particle (Shuujoshi) Ratio | 0.94 |
| Average Sentence Length | 0.79 | Particle Ratio | 0.91 |
| Normalized Dependency Distance | 0.72 | Marker Ratio | 0.56 |
| Maximum Dependency Tree Height | 0.69 | Noun Ratio | -0.34 |
| Maximum Center‑Embedding Depth | 0.59 | Mean Pairwise Sentence Distance | -0.29 |
| Mean Pairwise Sentence Distance | -0.40 | Object Ratio | -0.26 |
| Number of Morpheme | 0.37 | Case Particle (Kakujoshi) Ratio | -0.26 |
| Redundancy | 0.36 | Adjacent Perseveration Ratio | -0.23 |
| Vocabulary Ratio | -0.33 | Vocabulary Ratio | -0.23 |
| Density of Semantic Graph | -0.25 | Named Entity (TIME/DATE) Ratio | -0.19 |
| **Factor 3: Syntactic Complexity factor** | | **Factor 4: Pragmatic Engagement factor** | |

| **Factor5_feature** | **loadings** | **Factor6_feature** | **loadings** |
| --- | --- | --- | --- |
| Number of sentences | 0.88 | Mean Pairwise Word Similarity | 0.90 |
| Redundancy | 0.83 | Average Distance of Semantic Graph | -0.76 |
| Vocabulary Ratio | -0.81 | Variance of Pairwise Word Similarity | 0.75 |
| Number of Morphemes | 0.71 | Average Closeness Centrality of Semantic Graph | 0.59 |
| Density of Semantic Graph | -0.59 | Named Entity (LOCATION) Ratio | -0.47 |
| Global Duplicates Ratio | 0.55 | Density of Semantic Graph | 0.46 |
| Coordinating Conjunction Ratio | 0.46 | Diameter of Semantic Graph | -0.42 |
| Coordinating Conjunction Ratio (Syntax) | 0.45 | Prefix (Settouji) Ratio | 0.35 |
| Clustering Coefficient of Semantic Graph | -0.40 | Average Word Length | -0.33 |
| Pronoun Ratio | 0.40 | Determiner Ratio (Syntax) | -0.33 |
| **Factor 5: Amount of Speech factor** | | **Factor 6: Semantic Proximity factor** | |

| **Factor7_feature** | **loadings** | **Factor8_feature** | **loadings** |
| --- | --- | --- | --- |
| Case Particle (Kakujoshi) Ratio | -0.72 | Nominal Modifier Ratio | 0.76 |
| Adposition Ratio | -0.66 | Noun Ratio | 0.66 |
| Object Ratio | -0.62 | Suffix (Setsubiji) Ratio | 0.65 |
| Average Closeness Centrality of Semantic Graph | -0.42 | Proper Noun Ratio | 0.55 |
| Punctuation Ratio | 0.41 | Auxiliary Verb Ratio | -0.54 |
| Numeral Ratio | 0.36 | Compound Ratio | 0.52 |
| Adjacent Duplicates Ratio | 0.35 | Mean Pairwise Sentence Distance | 0.44 |
| Named Entity (TIME/DATE) Ratio | 0.35 | Named Entity (PERSON) Ratio | 0.39 |
| Clausal Modifier of Noun Ratio | -0.30 | Oblique Nominal Ratio | 0.33 |
| Adjacent Perseveration Ratio | 0.28 | Numeral Ratio | 0.33 |
| **Factor 7: Functional Simplicity factor** | | **Factor 8: Noun-related factor** | |

| **Factor9_feature** | **loadings** |
| --- | --- |
| Prenominal Adjective (Rentaishi) Ratio | 0.75 |
| Filler Interjection Ratio | 0.66 |
| Determiner Ratio | 0.49 |
| Adjective Ratio | -0.48 |
| Coordinating Conjunction Ratio | 0.46 |
| Coordinating Conjunction Ratio (Syntax) | 0.43 |
| Determiner Ratio (Syntax) | 0.35 |
| Normalized Center-Embedding Count | 0.35 |
| Average Word Length | -0.31 |
| Global Perseveration ratio | -0.29 |
| **Factor 9: Peripheral Modulation Factor** | |

Table S4. Varimax-rotated factor loading matrices for Tasks 1

**Task 2**

| **Factor1_feature** | **loadings** | **Factor2_feature** | **loadings** |
| --- | --- | --- | --- |
| Normalized Dependency Distance | -0.89 | Adverbial Modifier Ratio | -0.94 |
| Average Sentence Length | -0.86 | Adverb Ratio | -0.94 |
| Maximum Dependency Tree Height | -0.82 | Noun Ratio | 0.43 |
| Normalized Tree Height | 0.79 | Compound Ratio | 0.37 |
| Maximum Center‑Embedding Depth | -0.62 | Adjectival Modifier Ratio | -0.34 |
| Mean Pairwise Sentence Distance | 0.47 | Mean Pairwise Sentence Distance | 0.33 |
| Mean Adjacent Sentence Distance | 0.46 | Case Particle (Kakujoshi) Ratio | 0.33 |
| Number of Sentences | 0.45 | Adverbial Particle (Fukujoshi) Ratio | -0.30 |
| Variance of Pairwise Sentence Distance | -0.38 | Object Ratio | 0.27 |
| Case Particle (Kakujoshi) Ratio | -0.32 | Prefix (Settouji) Ratio | 0.26 |
| **Factor 1: Syntactic Complexity factor** | | **Factor 2: Modification-Coherence factor** | |

| **Factor3_feature** | **loadings** | **Factor4_feature** | **loadings** |
| --- | --- | --- | --- |
| Numeral Ratio | 0.95 | Redundancy | -0.91 |
| Numeric Modifier Ratio | 0.94 | Number of Morpheme | -0.86 |
| Named Entity (TIME/DATE) Ratio | 0.48 | Vocabulary Ratio | 0.80 |
| Adposition Ratio | 0.23 | Global Duplicates Ratio | -0.71 |
| Case Particle (Kakujoshi) Ratio | 0.20 | Number of Sentences | -0.68 |
| Adjective Ratio | 0.17 | Diameter of Semantic Graph | -0.51 |
| Global Duplicates Ratio | 0.16 | Average Distance of Semantic Graph | -0.44 |
| Auxiliary Verb Ratio | -0.16 | Maximum Center‑Embedding Depth | -0.39 |
| Case Marking Ratio | 0.16 | Focus Particle (Kakarijoshi) Ratio | -0.36 |
| Noun Ratio | 0.14 | Average Closeness Centrality of Semantic Graph | -0.35 |
| **Factor 3: Numeral Expression factor** | | **Factor 4: Lexical Density factor** | |

| **Factor5_feature** | **loadings** | **Factor6_feature** | **loadings** |
| --- | --- | --- | --- |
| Coordinating Conjunction Ratio (Syntax) | 0.94 | Particle Ratio | -0.92 |
| Coordinating Conjunction Ratio | 0.93 | Sentence-Final Particle (Shuujoshi) Ratio | -0.89 |
| Normalized Center‑Embedding Count | 0.43 | Marker Ratio | -0.80 |
| Determiner Ratio | 0.28 | Case Particle (Kakujoshi) Ratio | 0.46 |
| Auxiliary Verb Ratio | -0.25 | Proper Noun Ratio | -0.45 |
| Case Marking Ratio | -0.23 | Object Ratio | 0.45 |
| Average Word Length | -0.22 | Adverbial Particle (Fukujoshi) Ratio | -0.42 |
| Determiner Ratio (Syntax) | 0.21 | Noun Ratio | 0.41 |
| Nominal Subject Ratio | 0.21 | Adposition Ratio | 0.37 |
| General Interjection Ratio | -0.21 | Determiner Ratio | 0.32 |
| **Factor 5: Cohesion Marker factor** | | **Factor 6: Functional Marker Reduction Factor** | |

| **Factor7_feature** | **loadings** | **Factor8_feature** | **loadings** |
| --- | --- | --- | --- |
| Global Perseveration Ratio | 0.93 | Subordinating Conjunction Ratio | 0.85 |
| Adjacent Perseveration Ratio | 0.91 | Conjunctive Particle (Setuzokujoshi) Ratio | 0.81 |
| General Interjection Ratio | 0.48 | Verb Ratio | 0.54 |
| Mean Pairwise Word Similarity | 0.47 | Adverbial Clause Modifier Ratio | 0.52 |
| Compound Ratio | 0.41 | Marker Ratio | 0.37 |
| Interjection Ratio | 0.35 | Adjacent Duplicates Ratio | -0.32 |
| Mean Pairwise Sentence Distance | 0.30 | Clausal Modifier of Noun Ratio | -0.30 |
| Oblique Nominal Ratio | -0.27 | Sentence-Final Particle (Shuujoshi) Ratio | -0.27 |
| Clausal Modifier of Noun Ratio | -0.23 | Particle Ratio | -0.26 |
| Prenominal Adjective (Rentaishi) Ratio | -0.21 | Case Marking Ratio | -0.25 |
| **Factor 7: Perseveration factor** | | **Factor 8: Subordination factor** | |

| **Factor9_feature** | **loadings** | **Factor10_feature** | **loadings** |
| --- | --- | --- | --- |
| Density of Semantic Graph | 0.86 | Discourse Ratio | 0.81 |
| Average Closeness Centrality of Semantic Graph | 0.82 | Interjection Ratio | 0.78 |
| Clustering Coefficient of Semantic Graph | 0.82 | Punctuation Ratio | 0.73 |
| Average Distance of Semantic Graph | 0.76 | Adposition Ratio | -0.59 |
| Diameter of Semantic Graph | 0.70 | General Interjection Ratio | 0.59 |
| General Interjection Ratio | -0.31 | Nominal Subject Ratio | -0.51 |
| Normalized Center‑Embedding Count | 0.29 | Noun Ratio | -0.49 |
| Adposition Ratio | 0.28 | Verb Ratio | -0.47 |
| Average Word Length Ratio | -0.28 | Case Particle (Kakujoshi) Ratio | -0.46 |
| Maximum Center-Embedding Depth | 0.26 | Object Ratio | -0.44 |
| **Factor 9: Semantic Network factor** | | **Factor 10: Discourse factor** | |

| **Factor11_feature** | **loadings** |
| --- | --- |
| Auxiliary Verb Ratio | 0.67 |
| Case Marking Ratio | -0.58 |
| Variance of Pairwise Word Similarity | -0.48 |
| Adverbial Clause Modifier Ratio | 0.42 |
| Compound Ratio | -0.36 |
| Mean Adjacent Word Similarity | -0.35 |
| Prefix (Settouji) Ratio | -0.34 |
| Suffix (Setsubiji) Ratio | -0.33 |
| Noun Ratio | -0.33 |
| Average Word Length | 0.32 |
| **Factor 11: Syntactic–Morphological Imbalance Factor** | |

Table S5. Varimax-rotated factor loading matrices for Tasks 2

**Task 3**

| **Factor1_feature** | **loadings** | **Factor2_feature** | **loadings** |
| --- | --- | --- | --- |
| Adverb Ratio | 0.96 | Determiner Ratio | 0.98 |
| Adverbial Modifier Ratio | 0.93 | Determiner Ratio (Syntax) | 0.96 |
| Noun Ratio | -0.25 | Prenominal Adjective (Rentaishi) Ratio | 0.80 |
| Object Ratio | -0.22 | Mean Pairwise Sentence Distance | 0.18 |
| Case Particle (Kakujoshi) Ratio | -0.22 | General Interjection Ratio | -0.18 |
| Average Word Length | 0.22 | Variance of Adjacent Sentence Distance | 0.18 |
| Mean Adjacent Sentence Distance | -0.18 | Adverbial Clause Modifier Ratio | -0.18 |
| Compound Ratio | -0.17 | Normalized Center-Embedding Count | 0.17 |
| Mean Pairwise Sentence Distance | -0.17 | Suffix (Setsubiji) Ratio | 0.16 |
| Adposition Ratio | -0.17 | Filler Interjection Ratio | 0.16 |
| Factor 1: Modification factor | | Factor 2: Referential Function factor | |

| **Factor3_feature** | **loadings** | **Factor4_feature** | **loadings** |
| --- | --- | --- | --- |
| Numeral Ratio | -0.96 | Average Closeness Centrality of Semantic Graph | -0.89 |
| Numeric Modifier Ratio | -0.96 | Density of Semantic Graph | -0.85 |
| Suffix (Setsubiji) Ratio | -0.72 | Clustering Coefficient of Semantic Graph | -0.83 |
| Average Word Length | 0.24 | Average Distance of Semantic Graph | -0.73 |
| Noun Ratio | -0.23 | Diameter of Semantic Graph | -0.69 |
| Oblique Nominal Ratio | -0.20 | Adverbial Clause Modifier Ratio | 0.41 |
| Normalized Center-Embedding Count | -0.20 | Normalized Center-Embedding Count | -0.34 |
| Prefix (Settouji) Ratio | -0.20 | Vocabulary Ratio | 0.31 |
| Marker Ratio | 0.20 | Nominal Subject Ratio | 0.30 |
| Mean Pairwise Word Similarity | 0.19 | Average Word Length | 0.29 |
| Factor 3: Quantitative Expression factor | | Factor 4: Semantic Proximity factor | |

| **Factor5_feature** | **loadings** | **Factor6_feature** | **loadings** |
| --- | --- | --- | --- |
| Interjection Ratio | 0.97 | Case Particle (Kakujoshi) Ratio | 0.86 |
| Discourse Ratio | 0.96 | Adposition Ratio | 0.85 |
| General Interjection Ratio | 0.78 | Particle Ratio | -0.78 |
| Punctuation Ratio | 0.60 | Noun Ratio | 0.78 |
| Filler Interjection Ratio | 0.49 | Sentence-Final Particle (Shuujoshi) Ratio | -0.77 |
| Adjacent Perseveration Ratio | 0.48 | Object Ratio | 0.74 |
| Marker Ratio | -0.47 | Conjunctive Particle (Setuzokujoshi) Ratio | 0.61 |
| Global Perseveration Ratio | 0.46 | Auxilliary Verb Ratio | -0.60 |
| Case Marking Ratio | 0.45 | Pronoun Ratio | -0.60 |
| Oblique Nominal Ratio | -0.43 | Marker Ratio | -0.59 |
| Factor 5: Discourse factor | | Factor 6: Syntactic Function Word factor | |

| **Factor7_feature** | **loadings** | **Factor8_feature** | **loadings** |
| --- | --- | --- | --- |
| Redundancy Ratio | -0.91 | Average Sentence Length | 0.91 |
| Number of Morpheme | -0.90 | Normalized Dependency Distance | 0.84 |
| Vocabulary Ratio | 0.78 | Normalized Tree Height | -0.82 |
| Number of Sentence | -0.74 | Maximum Dependency Tree Height | 0.76 |
| Adjacent Duplicates Ratio | -0.58 | Maximum Center-Embedding Depth | 0.64 |
| Diameter of Semantic Graph | -0.48 | Number of Sentence | -0.47 |
| Average Word Length | 0.45 | Variance of Pairwise Sentence Distance | 0.41 |
| Mean Pairwise Word Similarity | 0.45 | Variance of Adjacent Sentence Distance | 0.27 |
| Average Distance of Semantic Graph | -0.42 | Case Particle (Kakujoshi) Ratio | 0.26 |
| Mean Adjacent Sentence Distance | 0.37 | Global Perseveration Ratio | -0.25 |
| Factor 7: Amount of Speech factor | | Factor 8: Syntactic Complexity factor | |

| **Factor9_feature** | **loadings** |
| --- | --- |
| Named Entity (PERSON) Ratio | 0.70 |
| Proper Noun Ratio | 0.64 |
| Pronoun Ratio | 0.55 |
| Focus Particle (Kakarijoshi) Ratio | 0.52 |
| Compound Ratio | 0.50 |
| Case Marking Ratio | 0.49 |
| Nominal Modifier Ratio | 0.47 |
| Variance of Adjacent Word Similarity | 0.44 |
| Verb Ratio | -0.44 |
| Adjacent Duplicates Ratio | 0.40 |
| Factor 9: Referential-Person factor | |

Table S6. Varimax-rotated factor loading matrices for Tasks 3

**S2-2-3. Representative feature tables**

**Task 1**

| **Factor** | **Feature** | **Loading** | **Absolute loading** | **Maximum cross-loading** | **Loading gap** |
| --- | --- | --- | --- | --- | --- |
| **Factor1** | Interjection Ratio | 0.93 | 0.93 | 0.21 | 0.73 |
| **Factor2** | Adverb Ratio | 0.95 | 0.95 | 0.18 | 0.77 |
| **Factor3** | Normalized Tree Height | -0.85 | 0.85 | 0.23 | 0.62 |
| **Factor4** | Sentence-Final Particle (Shuujoshi) Ratio | 0.94 | 0.94 | 0.17 | 0.76 |
| **Factor5** | Number of sentences | 0.88 | 0.88 | 0.22 | 0.67 |
| **Factor6** | Mean Pairwise Word Similarity | 0.90 | 0.90 | 0.15 | 0.76 |
| **Factor7** | Case Particle (Kakujoshi) Ratio | -0.72 | 0.72 | 0.44 | 0.28 |
| **Factor8** | Nominal Modifier Ratio | 0.76 | 0.76 | 0.26 | 0.50 |
| **Factor9** | Prenominal Adjective (Rentaishi) Ratio | 0.75 | 0.75 | 0.21 | 0.55 |

Table S7. Representative Feature by Factor and its selection log (Task 1).

**Task 2**

| **Factor** | **Feature** | **Loading** | **Absolute loading** | **Maximum cross-loading** | **Loading gap** |
| --- | --- | --- | --- | --- | --- |
| **Factor1** | Normalized Dependency Distance | -0.89 | 0.89 | 0.30 | 0.59 |
| **Factor2** | Adverbial Modifier Ratio | -0.94 | 0.94 | 0.12 | 0.82 |
| **Factor3** | Numeral Ratio | 0.95 | 0.95 | 0.15 | 0.79 |
| **Factor4** | Redundancy | -0.91 | 0.91 | 0.25 | 0.66 |
| **Factor5** | Coordinating Conjunction Ratio (Syntax) | 0.94 | 0.94 | 0.16 | 0.78 |
| **Factor6** | Particle Ratio | -0.92 | 0.92 | 0.26 | 0.66 |
| **Factor7** | Global Perseveration Ratio | 0.93 | 0.93 | 0.23 | 0.69 |
| **Factor8** | Subordinating Conjunction Ratio | 0.85 | 0.85 | 0.22 | 0.63 |
| **Factor9** | Density of Semantic Graph | 0.86 | 0.86 | 0.13 | 0.73 |
| **Factor10** | Discourse Ratio | 0.81 | 0.81 | 0.21 | 0.61 |
| **Factor11** | Auxiliary Verb Ratio | 0.67 | 0.67 | 0.25 | 0.42 |

Table S8. Representative Feature by Factor and its selection log (Task 2).

**Task 3**

| **Factor** | **Feature** | **Loading** | **Absolute loading** | **Maximum cross-loading** | **Loading gap** |
| --- | --- | --- | --- | --- | --- |
| **Factor1** | Adverb Ratio | 0.96 | 0.96 | 0.21 | 0.75 |
| **Factor2** | Determiner Ratio | 0.98 | 0.98 | 0.11 | 0.87 |
| **Factor3** | Numeral Ratio | -0.96 | 0.96 | 0.16 | 0.80 |
| **Factor4** | Average Closeness Centrality Ratio | -0.89 | 0.89 | 0.27 | 0.62 |
| **Factor5** | Interjection Ratio | 0.97 | 0.97 | 0.07 | 0.89 |
| **Factor6** | Case Particle (Kakujoshi) Ratio | 0.86 | 0.86 | 0.26 | 0.60 |
| **Factor7** | Redundancy | -0.91 | 0.91 | 0.18 | 0.73 |
| **Factor8** | Average Sentence Length | 0.91 | 0.91 | 0.21 | 0.69 |
| **Factor9** | Named Entity (PERSON) Ratio | 0.70 | 0.70 | 0.14 | 0.56 |

Table S9. Representative Feature by Factor and its selection log (Task 3).

**S2-3-4. Model Adequacy Diagnostics**

The adequacy of the Varimax-rotated factor models for Tasks 1–3 was assessed by comparing the empirical correlation matrix with the model-implied reproduced matrix. Across all tasks, the retained factor solutions captured the major correlational structure while leaving a subset of feature-specific dependencies unexplained.

- Task 1: The maximum absolute residual correlation was 0.58, and the Frobenius norm was 3.67.
- Task 2: The maximum absolute residual correlation was 0.54, and the Frobenius norm was 4.10.
- Task 3: The maximum absolute residual correlation was 0.70, and the Frobenius norm was 4.10.

For transparency, **Table S10-12** reports the feature pairs with the largest residual correlations for each task.

| **feature_i** | **feature_j** | **absolute residual correlation** | **residual correlation** | **raw correlation** |
| --- | --- | --- | --- | --- |
| Numeral Ratio | Numeric Modifier Ratio | 0.58 | 0.58 | 0.90 |
| Variance of Adjacent Sentence Distance | Variance of Pairwise Sentence Distance | 0.43 | 0.43 | 0.74 |
| Proper Noun Ratio | Named Entity (PERSON) Ratio | 0.42 | 0.42 | 0.70 |
| Determiner Ratio | Determiner Ratio (Syntax) | 0.32 | 0.32 | 0.69 |
| Proper Noun Ratio | Named Entity (LOCATION) Ratio | 0.27 | 0.27 | 0.60 |
| Focus Particle (Kakarijoshi) Ratio | Nominal Subject Ratio | 0.26 | 0.26 | 0.52 |
| Pronoun Ratio | Adverbial Particle (Fukujoshi) Ratio | 0.25 | 0.25 | 0.52 |
| Symbol Ratio | Named Entity (PERSON) Ratio | 0.25 | 0.25 | 0.34 |
| Adverbial Particle (Fukujoshi) Ratio | Oblique Nominal Ratio | 0.24 | 0.24 | 0.42 |
| Numeral Ratio | Named Entity (TIME/DATE) Ratio | 0.24 | 0.24 | 0.56 |
| Coordinating Conjunction Ratio | Coordinating Conjunction Ratio (Syntax) | 0.23 | 0.23 | 0.99 |
| Mean Adjacent Word Similarity | Variance of Adjacent Word Similarity | 0.23 | 0.23 | 0.29 |
| Named Entity (LOCATION) Ratio | Numeric Modifier Ratio | 0.22 | -0.22 | 0.05 |
| Proper Noun Ratio | Symbol Ratio | 0.22 | 0.22 | 0.33 |
| Variance of Pairwise Sentence Distance | Focus Particle (Kakarijoshi) Ratio | 0.22 | -0.22 | -0.35 |
| Adjective Ratio | Overridden Disfluency Ratio | 0.21 | -0.21 | -0.21 |
| Pronoun Ratio | Oblique Nominal Ratio | 0.21 | 0.21 | 0.57 |
| Average Word Length | Named Entity (PERSON) Ratio | 0.20 | 0.20 | 0.07 |
| Adjective Ratio | Numeric Modifier Ratio | 0.20 | -0.20 | -0.21 |
| Proper Noun Ratio | Numeric Modifier Ratio | 0.20 | -0.20 | 0.08 |
| Adverbial Particle (Fukujoshi) Ratio | Focus Particle (Kakarijoshi) Ratio | 0.20 | -0.20 | -0.04 |
| Mean Adjacent Sentence Distance | Variance of Adjacent Sentence Distance | 0.20 | -0.20 | -0.45 |
| Average Distance of Semantic Graph | Diameter of Semantic Graph | 0.20 | 0.20 | 0.61 |
| Adjacent Perseveration Ratio | Global Perseveration Ratio | 0.19 | 0.19 | 0.93 |
| Mean Adjacent Sentence Distance | Mean Pairwise Sentence Distance | 0.19 | 0.19 | 0.67 |
| Variance of Pairwise Sentence Distance | Adjective Ratio | 0.19 | -0.19 | -0.17 |
| Mean Adjacent Word Similarity | Clausal Subject Ratio | 0.18 | 0.18 | 0.19 |
| Diameter of Semantic Graph | Symbol Ratio | 0.18 | 0.18 | 0.19 |
| Variance of Adjacent Sentence Distance | Focus Particle (Kakarijoshi) Ratio | 0.18 | -0.18 | -0.31 |
| Auxiliary Verb Ratio | Case Marking Ratio | 0.17 | -0.17 | -0.59 |

**Table S10:** the feature pairs with the largest residual correlations for Task 1.

| **feature_i** | **feature_j** | **absolute residual correlation** | **residual correlation** | **raw correlation** |
| --- | --- | --- | --- | --- |
| Proper Noun Ratio | Named Entity (PERSON) Ratio | 0.54 | 0.54 | 0.63 |
| Determiner Ratio | Determiner Ratio (Syntax) | 0.49 | 0.49 | 0.88 |
| Mean Adjacent Word Similarity | Variance of Adjacent Word Similarity | 0.47 | 0.47 | 0.56 |
| Variance of Adjacent Sentence Distance | Variance of Pairwise Sentence Distance | 0.46 | 0.46 | 0.61 |
| Determiner Ratio | Prenominal Adjective (Rentaishi) Ratio | 0.39 | 0.39 | 0.72 |
| Adverbial Particle (Fukujoshi) Ratio | Oblique Nominal Ratio | 0.32 | 0.32 | 0.27 |
| Prenominal Adjective (Rentaishi) Ratio | Determiner Ratio | 0.31 | 0.31 | 0.57 |
| Pronoun Ratio | Adverbial Particle (Fukujoshi) Ratio | 0.30 | 0.30 | 0.62 |
| Pronoun Ratio | Oblique Nominal Ratio | 0.28 | 0.28 | 0.23 |
| Mean Adjacent Sentence Distance | Mean Pairwise Sentence Distance | 0.27 | 0.27 | 0.63 |
| Variance of Adjacent Sentence Distance | Adjacent Duplicates Ratio | 0.26 | 0.26 | 0.23 |
| Average Word Length | Prenominal Adjective (Rentaishi) Ratio | 0.26 | -0.26 | -0.40 |
| Mean Adjacent Sentence Distance | Named Entity (LOCATION) Ratio | 0.24 | 0.24 | 0.22 |
| Variance of Adjacent Sentence Distance | Pronoun Ratio | 0.24 | -0.24 | -0.30 |
| Mean Adjacent Word Similarity | Named Entity (DEITY) Ratio | 0.23 | -0.23 | -0.27 |
| Prefix (Settouji) Ratio | Suffix (Setsubiji) Ratio | 0.22 | 0.22 | 0.52 |
| Suffix (Setsubiji) Ratio | Nominal Modifier Ratio | 0.22 | -0.22 | -0.04 |
| Mean Pairwise Word Similarity | Variance of Pairwise Word Similarity | 0.21 | 0.21 | 0.42 |
| Adverbial Particle (Fukujoshi) Ratio | Nominal Modifier Ratio | 0.21 | -0.21 | -0.34 |
| Variance of Pairwise Sentence Distance | Oblique Nominal Ratio | 0.21 | -0.21 | -0.12 |
| General Interjection Ratio | Filler Interjection Ratio | 0.21 | -0.21 | -0.18 |
| Named Entity (LOCATION) Ratio | Nominal Modifier Ratio | 0.20 | 0.20 | 0.23 |
| Suffix (Setsubiji) Ratio | Compound Ratio | 0.20 | 0.20 | 0.44 |
| Adjective Ratio | Suffix (Setsubiji) Ratio | 0.20 | 0.20 | 0.14 |
| General Interjection Ratio | Prenominal Adjective (Rentaishi) Ratio | 0.20 | -0.20 | -0.34 |
| Average Word Length | Proper Noun Ratio | 0.20 | 0.20 | 0.22 |
| Clausal Modifier of Noun Ratio | Nominal Modifier Ratio | 0.20 | -0.20 | 0.00 |
| Adjacent Duplicates Ratio | Oblique Nominal Ratio | 0.19 | -0.19 | -0.34 |
| Average Word Length | Adjective Ratio | 0.19 | 0.19 | 0.28 |
| Average Word Length | Named Entity (PERSON) Ratio | 0.19 | 0.19 | 0.24 |

**Table S11:** the feature pairs with the largest residual correlations for Task 2.

| **feature_i** | **feature_j** | **absolute residual correlation** | **residual correlation** | **raw correlation** |
| --- | --- | --- | --- | --- |
| Coordinating Conjunction Ratio | Coordinating Conjunction Ratio (Syntax) | 0.70 | 0.70 | 0.98 |
| Mean Adjacent Word Similarity | Variance of Adjacent Word Similarity | 0.58 | 0.58 | 0.72 |
| Adjacent Perseveration Ratio | Global Perseveration Ratio | 0.58 | 0.58 | 0.97 |
| Subordinating Conjunction Ratio | Conjunctive Particle (Setuzokujoshi) Ratio | 0.47 | 0.47 | 0.81 |
| Prefix (Settouji) Ratio | Compound Ratio | 0.41 | 0.41 | 0.47 |
| Variance of Adjacent Sentence Distance | Variance of Pairwise Sentence Distance | 0.36 | 0.36 | 0.55 |
| Proper Noun Ratio | Named Entity (PERSON) Ratio | 0.34 | 0.34 | 0.83 |
| Symbol Ratio | Overridden Disfluency Ratio | 0.33 | 0.33 | 0.35 |
| General Interjection Ratio | Filler Interjection Ratio | 0.33 | -0.33 | -0.07 |
| Subordinating Conjunction Ratio | Marker Ratio | 0.27 | 0.27 | 0.32 |
| Mean Adjacent Sentence Distance | Mean Pairwise Sentence Distance | 0.27 | 0.27 | 0.61 |
| Auxiliary Verb Ratio | Conjunctive Particle (Setuzokujoshi) Ratio | 0.26 | -0.26 | -0.54 |
| Clausal Modifier of Noun Ratio | Adverbial Clause Modifier Ratio | 0.25 | -0.25 | -0.22 |
| Adjective Ratio | Adjectival Modifier Ratio | 0.25 | 0.25 | 0.20 |
| Variance of Pairwise Sentence Distance | Named Entity (LOCATION) Ratio | 0.24 | 0.24 | 0.30 |
| Auxiliary Verb Ratio | Subordinating Conjunction Ratio | 0.24 | -0.24 | -0.34 |
| Variance of Pairwise Word Similarity | Prefix (Settouji) Ratio | 0.23 | 0.23 | 0.25 |
| Subordinating Conjunction Ratio | Clausal Modifier of Noun Ratio | 0.22 | -0.22 | -0.08 |
| Verb Ratio | Conjunctive Particle (Setuzokujoshi) Ratio | 0.22 | 0.22 | 0.67 |
| Particle Ratio | Sentence-Final Particle (Shuujoshi) Ratio | 0.22 | 0.22 | 0.95 |
| Mean Adjacent Word Similarity | Variance of Adjacent Sentence Distance | 0.22 | -0.22 | -0.13 |
| Mean Pairwise Word Similarity | Variance of Pairwise Word Similarity | 0.21 | 0.21 | 0.52 |
| Adjective Ratio | Coordinating Conjunction Ratio | 0.20 | -0.20 | -0.19 |
| Variance of Adjacent Sentence Distance | Clausal Subject Ratio | 0.20 | -0.20 | -0.17 |
| Adjective Ratio | Coordinating Conjunction Ratio (Syntax) | 0.20 | -0.20 | -0.17 |
| Focus Particle (Kakarijoshi) Ratio | Nominal Subject Ratio | 0.20 | 0.20 | 0.31 |
| Conjunctive Particle (Setuzokujoshi) Ratio | Marker Ratio | 0.19 | 0.19 | 0.00 |
| Adjacent Perseveration Ratio | Filler Interjection Ratio | 0.19 | -0.19 | -0.07 |
| Proper Noun Ratio | Compound Ratio | 0.18 | 0.18 | 0.55 |
| Global Perseveration Ratio | General Interjection Ratio | 0.18 | 0.18 | 0.68 |

**Table S12:** the feature pairs with the largest residual correlations for Task 3.

**S2-3-5. Factor stability.**

Factor-solution and representative-feature stability were assessed using two resampling procedures (bootstrap: n=2,000; Monte-Carlo subsampling: n=2,000, 70%). For each resample, the full pipeline (PA → FA → Varimax → representative selection) was re-estimated and Procrustes-aligned to the original solution. Tucker’s factor congruence values indicated high reproducibility across tasks. The top-5 representative-feature selection frequencies for each factor are listed in Tables S13–15, and the corresponding stability visualizations are shown in Figures S7–9.

**
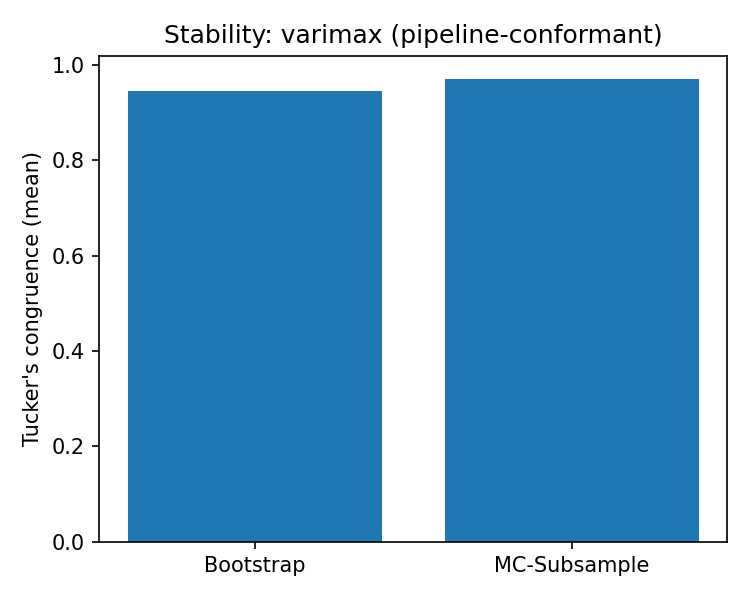
**

**Figure S7. Stability of Varimax-Rotated Factor Solutions (Task 1)**

Mean Tucker’s factor congruence coefficients from 2,000 bootstrap samples and 2,000 Monte-Carlo subsamples (70%). Both procedures showed high reproducibility (bootstrap: mean = 0.94, SD = 0.02; MC-subsample: mean = 0.97, SD = 0.02).

| **Factor1_feature** | **freq** | **Factor2_feature** | **freq** | **Factor3_feature** | **freq** |
| --- | --- | --- | --- | --- | --- |
| **Interjection Ratio** | **0.411** | **Adverb Ratio** | **0.827** | **Normalized Tree Height** | **0.887** |
| **Discourse Ratio** | **0.404** | **Adverb Modifier Ratio** | **0.170** | **Average Sentence Length** | **0.103** |
| **Global Perseveration Ratio** | **0.088** | **Prefix (Settouji) Ratio** | **0.002** | **Maximum Tree Height** | **0.006** |
| **General Interjection Ratio** | **0.052** | **Mean Pairwise Sentence Distance** | **0.001** | **Numeric Modifier Ratio** | **0.001** |
| **Subordinating Conjunction Ratio** | **0.026** | **Named Entity (PERSON) Ratio** | **0.001** | **Proper Noun Ratio** | **0.001** |

| **Factor4_feature** | **freq** | **Factor5_feature** | **freq** | **Factor6_feature** | **freq** |
| --- | --- | --- | --- | --- | --- |
| **Sentence-Final Particle (Shuujoshi) Ratio** | **0.742** | **Number of Sentence** | **0.759** | **Mean Pairwise Word Similarity** | **0.937** |
| **Particle Ratio** | **0.236** | **Redundancy** | **0.145** | **Average Distance of Semantic Graph** | **0.026** |
| **Adverbial Particle (Fukujoshi) Ratio** | **0.005** | **Vocabulary Ratio** | **0.096** | **Variance of Pairwise Word Similarity** | **0.015** |
| **Symbol Ratio** | **0.004** | **Coordinating Conjunction Ratio** | **0.001** | **Numeral Ratio** | **0.007** |
| **Numeric Modifier Ratio** | **0.004** |  |  | **Numeric Modifier Ratio** | **0.006** |

| **Factor7_feature** | **freq** | **Factor8_feature** | **freq** | **Factor9_feature** | **freq** |
| --- | --- | --- | --- | --- | --- |
| **Case Particle (Kakujoshi) Ratio** | **0.680** | **Nominal Modifier Ratio** | **0.606** | **Prenominal Adjective (Rentaishi) Ratio** | **0.592** |
| **Object Ratio** | **0.203** | **Proper Noun Ratio** | **0.149** | **Coordinating Conjunction Ratio** | **0.129** |
| **Numeral Ratio** | **0.040** | **Suffix (Setsubiji) Ratio** | **0.061** | **Filler Interjection Ratio** | **0.118** |
| **Numeric Modifier Ratio** | **0.032** | **Auxiliary Verb Ratio** | **0.054** | **Adjective Ratio** | **0.082** |
| **Adposition Ratio** | **0.031** | **Numeral Ratio** | **0.043** | **Determiner Ratio** | **0.043** |

Tables S13. Selection Frequencies of Representative Features (Task 1)
Top five representative features per factor ranked by selection frequency across resampling iterations.

**
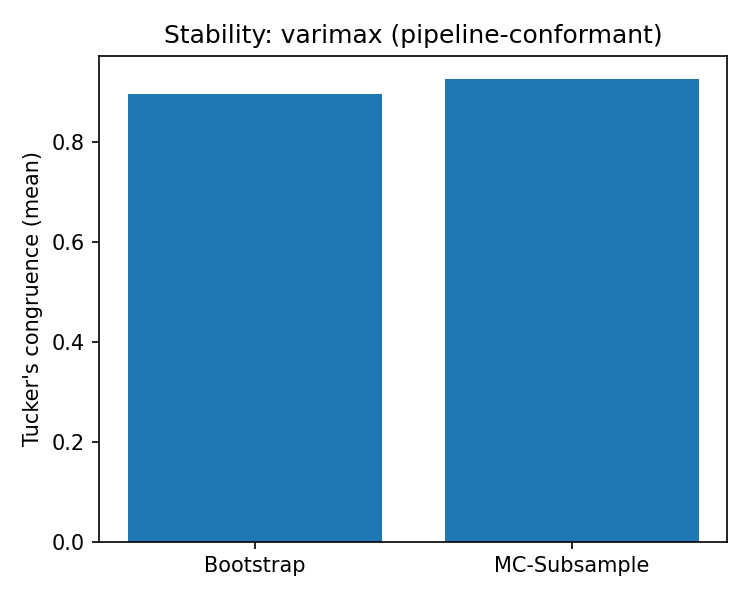
**

**Figure S8. Stability of Varimax-Rotated Factor Solutions (Task 2)**

Mean Tucker’s factor congruence coefficients from 2,000 bootstrap samples and 2,000 Monte-Carlo subsamples (70%). Both procedures showed high reproducibility (bootstrap: mean = 0.90, SD = 0.029; MC-subsample: mean = 0.93, SD = 0.026).

| **Factor1_feature** | **freq** | **Factor2_feature** | **freq** | **Factor3_feature** | **freq** |
| --- | --- | --- | --- | --- | --- |
| **Normalized Dependency Distance** | **0.46** | **Particle Ratio** | **0.84** | **Numeric Modifier Ratio** | **0.29** |
| **Average Sentence Length** | **0.34** | **Sentence-Final Particle (Shuujoshi) Ratio** | **0.16** | **Numeral Ratio** | **0.27** |
| **Normalized Tree Height** | **0.18** | **Marker Ratio** | **0.01** | **Filler Interjection Ratio** | **0.09** |
| **Maximum Dependency Tree Height** | **0.03** | **Overridden Disfluency Ratio** | **0.00** | **Prenominal Adjective (Rentaishi) Ratio** | **0.08** |
| **-** | **-** | **-** | **-** | **Named Entity (TIME/DATE) Ratio** | **0.05** |

| **Factor4_feature** | **freq** | **Factor5_feature** | **freq** | **Factor6_feature** | **freq** |
| --- | --- | --- | --- | --- | --- |
| **Redundancy** | **0.78** | **Coordinating Conjunction Ratio (Syntax)** | **0.42** | **Adverbial Modifier Ratio** | **0.54** |
| **Number of Morpheme** | **0.19** | **Coordinating Conjunction Ratio** | **0.37** | **Adverb Ratio** | **0.36** |
| **Vocabulary Ratio** | **0.03** | **Determiner Ratio** | **0.11** | **Pronoun Ratio** | **0.04** |
| **Number of Sentence** | **0.00** | **Prenominal Adjective (Rentaishi) Ratio** | **0.02** | **Adverbial Particle (Fukujoshi) Ratio** | **0.02** |
| **-** | **-** | **Normalized Center-Embedding Count** | **0.02** | **Determiner Ratio** | **0.01** |

| **Factor7_feature** | **freq** | **Factor8_feature** | **freq** | **Factor9_feature** | **freq** |
| --- | --- | --- | --- | --- | --- |
| **Adjacent Perseveration Ratio** | **0.33** | **Subordinating Conjunction Ratio** | **0.79** | **Density of Semantic Graph** | **0.59** |
| **Global Perseveration Ratio** | **0.27** | **Conjunctive Particle (Setuzokujoshi) Ratio** | **0.18** | **Average Closeness Centrality of Semantic Graph** | **0.20** |
| **Named Entity (DEITY) Ratio** | **0.09** | **Adverbial Clause Modifier Ratio** | **0.01** | **Clustering Coefficient of Semantic Graph** | **0.17** |
| **Oblique Nominal Ratio** | **0.05** | **Verb Ratio** | **0.01** | **Average Distance of Semantic Graph** | **0.04** |
| **General Interjection Ratio** | **0.04** | **Named Entity (LOCATION) Ratio** | **0.00** | **Auxiliary Verb Ratio** | **0.00** |

| **Factor10_feature** | **freq** | **Factor11_feature** | **freq** |
| --- | --- | --- | --- |
| **Discourse Ratio** | **0.67** | **Auxiliary Verb Ratio** | **0.43** |
| **Punctuation Ratio** | **0.17** | **Case Marking Ratio** | **0.18** |
| **Interjection Ratio** | **0.16** | **Variance of Pairwise Word Similarity** | **0.12** |
| **Nominal Subject Ratio** | **0.01** | **Suffix (Setsubiji) Ratio** | **0.06** |
| **Proper Noun Ratio** | **0.00** | **Prefix (Settouji) Ratio** | **0.05** |

Tables S14. Selection Frequencies of Representative Features (Task 2)
Top five representative features per factor ranked by selection frequency across resampling iterations.

**
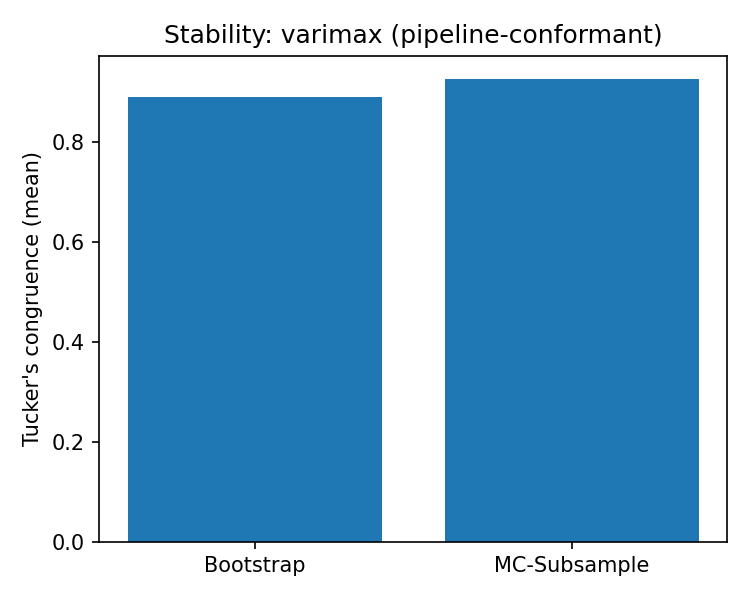
**

**Figure S9. Stability of Varimax-Rotated Factor Solutions (Task 3)**

Mean Tucker’s factor congruence coefficients from 2,000 bootstrap samples and 2,000 Monte-Carlo subsamples (70%). Both procedures showed high reproducibility (bootstrap: mean = 0.89, SD = 0.038; MC-subsample: mean = 0.93, SD = 0.04).

| **Factor1_feature** | **freq** | **Factor2_feature** | **freq** | **Factor3_feature** | **freq** |
| --- | --- | --- | --- | --- | --- |
| **Adverb Ratio** | **0.505** | **Determiner Ratio** | **0.688** | **Numeric Modifier Ratio** | **0.477** |
| **Adverbial Modifier Ratio** | **0.113** | **Determiner Ratio (Syntax)** | **0.223** | **Numeral Ratio** | **0.345** |
| **Subordinating Conjunction Ratio** | **0.082** | **Subordinating Conjunction Ratio** | **0.030** | **Subordinating Conjunction Ratio** | **0.101** |
| **Coordinating Conjunction Ratio (Syntax)** | **0.056** | **Clausal Modifier of Noun Ratio** | **0.010** | **Suffix (Setsubiji) Ratio** | **0.054** |
| **Clausal Modifier of Noun Ratio** | **0.050** | **Coordinating Conjunction Ratio** | **0.010** | **Global Perseveration Ratio** | **0.010** |

| **Factor4_feature** | **freq** | **Factor5_feature** | **freq** | **Factor6_feature** | **freq** |
| --- | --- | --- | --- | --- | --- |
| **Average Closeness Centrality of Semantic Graph** | **0.730** | **Discourse Ratio** | **0.493** | **Case Particle (Kakujoshi) Ratio** | **0.365** |
| **Density of Semantic Graph** | **0.169** | **Interjection Ratio** | **0.489** | **Adposition Ratio** | **0.297** |
| **Clustering Coefficient of Semantic Graph** | **0.093** | **General Interjection Ratio** | **0.012** | **Particle Ratio** | **0.111** |
| **Global Perseveration Ratio** | **0.003** | **Global Perseveration Ratio** | **0.005** | **Sentence-Final Particle (Shuujoshi) Ratio** | **0.107** |
| **Adjacent Perseveration Ratio** | **0.003** | **Adjacent Perseveration Ratio** | **0.002** | **Noun Ratio** | **0.092** |

| **Factor7_feature** | **freq** | **Factor8_feature** | **freq** | **Factor9_feature** | **freq** |
| --- | --- | --- | --- | --- | --- |
| **Redundancy** | **0.589** | **Average Sentence Length** | **0.861** | **Named Entity (PERSON) Ratio** | **0.563** |
| **Number of Morpheme** | **0.381** | **Normalized Dependency Distance** | **0.090** | **Subordinating Conjunction Ratio** | **0.099** |
| **Vocabulary Ratio** | **0.028** | **Normalized Tree Height** | **0.043** | **Variance of Adjacent Word Similarity** | **0.075** |
| **Number of Sentence** | **0.003** | **Maximum Dependency Tree Heigh** | **0.006** | **Coordinating Conjunction Ratio (Syntax)** | **0.073** |
| **-** | **-** | **Variance of Pairwise Sentence Distance** | **0.001** | **Proper Noun Ratio** | **0.037** |

Tables S15. Selection Frequencies of Representative Features (Task 3)
Top five representative features per factor ranked by selection frequency across resampling iterations.

**S2-3-6. Summary of Representative Features and Factor Interpretations**

Tables S16–S18 list the final representative features for each task (feature name, factor, loading). These are the features used in all subsequent GEE and diagnostic analyses.

| **Factor** | **Factor's interpretation** | **Representative Feature** | **Loading** |
| --- | --- | --- | --- |
| **Factor1** | **Discourse factor** | Interjection Ratio | 0.93 |
| **Factor2** | **Modification-Coherence factor** | Adverb Ratio | 0.95 |
| **Factor3** | **Syntactic Complexity factor** | Normalized Tree Height | -0.85 |
| **Factor4** | **Pragmatic Engagement factor** | Sentence-Final Particle (Shuujoshi) Ratio | 0.94 |
| **Factor5** | **Amount of Speech factor** | Number of sentences | 0.88 |
| **Factor6** | **Semantic Proximity factor** | Mean Pairwise Word Similarity | 0.90 |
| **Factor7** | **Functional Simplicity factor** | Case Particle (Kakujoshi) Ratio | -0.72 |
| **Factor8** | **Noun-related factor** | Nominal Modifier Ratio | 0.76 |
| **Factor9** | **Peripheral Modulation factor** | Prenominal Adjective (Rentaishi) Ratio | 0.75 |

Tables S16. Representative Features and Factor Interpretations for Task 1

This table lists the representative feature selected for each factor in Task 1, along with the assigned factor interpretation and the corresponding Varimax loading. These representatives were used in the subsequent GEE and diagnostic analyses. Tables S17 and S18 provide the corresponding results for Tasks 2 and 3.

| **Factor** | **Factor's interpretation** | **Feature** | **Loading** |
| --- | --- | --- | --- |
| **Factor1** | **Syntactic Complexity factor** | Normalized Dependency Distance | -0.89 |
| **Factor2** | **Modification-Coherence factor** | Adverbial Modifier Ratio | -0.94 |
| **Factor3** | **Numeral Expression factor** | Numeral Ratio | 0.95 |
| **Factor4** | **Lexical Diversity factor** | Redundancy | -0.91 |
| **Factor5** | **Cohesion Marker factor** | Coordinating Conjunction Ratio (Syntax) | 0.94 |
| **Factor6** | **Functional Marker Reduction factor** | Particle Ratio | -0.92 |
| **Factor7** | **Perseveration factor** | Global Perseveration Ratio | 0.93 |
| **Factor8** | **Subordination factor** | Subordinating Conjunction Ratio | 0.85 |
| **Factor9** | **Semantic Network factor** | Density of Semantic Graph | 0.86 |
| **Factor10** | **Discourse factor** | Discourse Ratio | 0.81 |
| **Factor11** | **Syntactic-Morphological Imbalance factor** | Auxiliary Verb Ratio | 0.67 |

Tables S17. Representative Features and Factor Interpretations for Task 2

| **Factor** | **Factor's interpretation** | **Feature** | **Loading** |
| --- | --- | --- | --- |
| **Factor1** | **Modification factor** | Adverb Ratio | 0.96 |
| **Factor2** | **Referential Function factor** | Determiner Ratio | 0.98 |
| **Factor3** | **Quantitative Expression factor** | Numeral Ratio | -0.96 |
| **Factor4** | **Semantic Proximity factor** | Average Closeness Centrality of Semantic Graph | -0.89 |
| **Factor5** | **Discourse factor** | Interjection Ratio | 0.97 |
| **Factor6** | **Syntactic Function Word factor** | Case Particle (Kakujoshi) Ratio | 0.86 |
| **Factor7** | **Amount of Speech factor** | Redundancy | -0.91 |
| **Factor8** | **Syntactic Complexity factor** | Average Sentence Length | 0.91 |
| **Factor9** | **Referential-Person factor** | Named Entity (PERSON) Ratio | 0.70 |

Tables S18. Representative Features and Factor Interpretations for Task 3

**S2-4. Task-specific Validation & Predictive Performance**

**Task 1**

Table S19 summarizes the task-specific GEE results. Several linguistic features showed significant associations with diagnosis after FDR correction. Mean Pairwise Word Similarity was positively associated with schizophrenia status (β = 0.83, OR = 2.30), whereas Adverb Ratio (β = −1.40, OR = 0.25) and Case Particle (Kakujoshi) Ratio (β = −1.08, OR = 0.34) were negatively associated. Age and most other syntactic variables were not significant.

| **Variable** | **Beta** | **SE** | **z** | **95%CI** | **OR** | **OR_CI** | **p** | **FDR_p** |
| --- | --- | --- | --- | --- | --- | --- | --- | --- |
| **Age** | **-0.10** | **0.23** | **-0.44** | **[-0.556 – 0.353]** | **0.90** | **[0.573 – 1.424]** | **0.662** | **0.809** |
| **Gender (male=1)** | **-1.00** | **0.51** | **-1.95** | **[-2.000 – 0.005]** | **0.37** | **[0.135 – 1.005]** | **0.051** | **0.140** |
| **Number of Sentences** | **0.03** | **0.20** | **0.17** | **[-0.360 – 0.428]** | **1.04** | **[0.698 – 1.534]** | **0.866** | **0.866** |
| **Mean Pairwise Word Similarity** | **0.83** | **0.23** | **3.70** | **[0.391 – 1.274]** | **2.30** | **[1.479 – 3.577]** | **<0.001** | **<0.01** |
| **Adverb Ratio** | **-1.40** | **0.30** | **-4.72** | **[-1.984 – -0.819]** | **0.25** | **[0.138 – 0.441]** | **<0.001** | **<0.01** |
| **Interjection Ratio** | **-0.17** | **0.26** | **-0.63** | **[-0.678 – 0.348]** | **0.85** | **[0.508 – 1.416]** | **0.529** | **0.727** |
| **Case Particles (Kakujoshi) Ratio** | **-1.08** | **0.28** | **-3.85** | **[-1.637 – -0.532]** | **0.34** | **[0.195 – 0.588]** | **<0.001** | **<0.01** |
| **Sentence-final Particles (Shuujoshi) Ratio** | **-0.18** | **0.25** | **-0.72** | **[-0.663 – 0.305]** | **0.84** | **[0.515 – 1.357]** | **0.469** | **0.727** |
| **Prenominal Adjective Ratio** | **0.31** | **0.19** | **1.64** | **[-0.061 – 0.675]** | **1.36** | **[0.941 – 1.965]** | **0.102** | **0.224** |
| **Nominal Modifier Ratio** | **0.14** | **0.22** | **0.65** | **[-0.288 – 0.573]** | **1.15** | **[0.750 – 1.773]** | **0.516** | **0.727** |
| **Tree Height Ratio** | **-0.04** | **0.21** | **-0.20** | **[-0.460 – 0.376]** | **0.96** | **[0.631 – 1.457]** | **0.844** | **0.866** |
| **Constant** | **1.00** | **0.36** | **2.83** | **[0.308 – 1.700]** | **2.73** | **[1.361 – 5.474]** | **0.005** |  |

Tables S19. Task 1 GEE results using representative linguistic features

This table summarizes the Task-1 GEE model using the representative features. Coefficients are shown as β, odds ratios (OR), 95% confidence intervals, p-values, and FDR-adjusted p-values. Significant associations after FDR correction (FDR < 0.05) are highlighted.

**Predictive Performance**

Predictive performance for Task 1 was evaluated on the held-out test set, with all confidence intervals obtained via percentile bootstrap resampling (2,000 iterations). The classifier showed stable and robust out-of-sample discrimination, with performance metrics summarized in Table S20.

| **Metric** | **Estimate** | **95% CI (Lower)** | **95% CI (Upper)** |
| --- | --- | --- | --- |
| **AUC** | **0.869** | **0.743** | **0.969** |
| **Sensitivity** | **0.762** | **0.565** | **0.941** |
| **Specificity** | **0.9** | **0.75** | **1** |
| **PPV** | **0.889** | **0.711** | **1** |
| **NPV** | **0.783** | **0.591** | **0.947** |
| **Accuracy** | **0.829** | **0.707** | **0.927** |
| **F1** | **0.821** | **0.667** | **0.936** |
| **Balanced Accuracy** | **0.831** | **0.709** | **0.939** |

Table S20. Predictive Performance of the Task 1 Classifier

This table summarizes the out-of-sample predictive performance of the Task 1 GEE model, with 95% confidence intervals estimated via percentile bootstrap resampling (2,000 iterations). Metrics include discrimination, calibration-independent accuracy indices, and threshold-based performance. AUC = area under the ROC curve; PPV = positive predictive value; NPV = negative predictive value.

**Task 2**

Table S21 summarizes the task-specific GEE results. Several linguistic features showed significant associations with diagnosis after FDR correction. Redundancy and Adverbial Modifier Ratio were negatively associated with schizophrenia status, indicating that reduced linguistic economy and fewer adverbial modifications characterized patient speech in this narrative task. Discourse Ratio also showed a negative association, whereas Global Perseveration Ratio showed a strong positive association with schizophrenia. Age, gender, and most other syntactic or lexical variables were not significant.

| **Variable** | **Beta** | **SE** | **z** | **95%CI** | **OR** | **OR_CI** | **p** | **FDR_p** |
| --- | --- | --- | --- | --- | --- | --- | --- | --- |
| Age | -0.25 | 0.22 | -1.14 | [-0.674 – 0.178] | 0.78 | [0.510 – 1.195] | 0.25 | 0.41 |
| Gender (female=1) | -0.63 | 0.41 | -1.51 | [-1.437 – 0.186] | 0.54 | [0.238 – 1.205] | 0.13 | 0.24 |
| Redundancy | -0.43 | 0.18 | -2.40 | [-0.788 – -0.079] | 0.65 | [0.455 – 0.924] | 0.02 | 0.04 |
| Density of Semantic Graph | -0.21 | 0.14 | -1.52 | [-0.479 – 0.061] | 0.81 | [0.619 – 1.062] | 0.13 | 0.24 |
| Numeral Ratio | 0.14 | 0.16 | 0.92 | [-0.161 – 0.445] | 1.15 | [0.851 – 1.560] | 0.36 | 0.47 |
| Auxiliary Verb Ratio | -0.05 | 0.18 | -0.30 | [-0.400 – 0.293] | 0.95 | [0.670 – 1.340] | 0.76 | 0.76 |
| Subordinating Conjunction Ratio | -0.19 | 0.19 | -1.02 | [-0.563 – 0.179] | 0.83 | [0.569 – 1.196] | 0.31 | 0.45 |
| Particle Ratio | 0.13 | 0.20 | 0.67 | [-0.250 – 0.513] | 1.14 | [0.779 – 1.670] | 0.50 | 0.54 |
| Adverbial Modifier Ratio | -0.39 | 0.16 | -2.45 | [-0.707 – -0.079] | 0.68 | [0.493 – 0.924] | 0.01 | 0.04 |
| Coordinating Conjunction Ratio | -0.15 | 0.18 | -0.80 | [-0.506 – 0.212] | 0.86 | [0.603 – 1.236] | 0.42 | 0.50 |
| Discourse Ratio | -0.70 | 0.24 | -2.85 | [-1.174 – -0.217] | 0.50 | [0.309 – 0.805] | 0.00 | 0.03 |
| Global Perseveration Ratio | 1.63 | 0.49 | 3.37 | [0.681 – 2.581] | 5.11 | [1.976 – 13.216] | 0.00 | 0.01 |
| Normalized Dependency Distance | -0.56 | 0.23 | -2.41 | [-1.010 – -0.104] | 0.57 | [0.364 – 0.901] | 0.02 | 0.04 |
| Constant | 0.82 | 0.31 | 2.69 | [0.223 – 1.418] | 2.27 | [1.250 – 4.130] | 0.01 |  |

Table S21. Task 2 GEE results using representative linguistic features

**Predictive Performance**

| **Metric** | **Estimate** | **95% CI (Lower)** | **95% CI (Upper)** |
| --- | --- | --- | --- |
| **AUC** | **0.805** | **0.661** | **0.923** |
| **Sensitivity** | **0.667** | **0.452** | **0.854** |
| **Specificity** | **0.75** | **0.548** | **0.941** |
| **PPV** | **0.737** | **0.5** | **0.935** |
| **NPV** | **0.682** | **0.476** | **0.864** |
| **Accuracy** | **0.707** | **0.561** | **0.829** |
| **F1** | **0.7** | **0.5** | **0.842** |
| **Balanced Accuracy** | **0.708** | **0.561** | **0.837** |

Table S22. Predictive Performance of the Task 2 Classifier

**Task 3**

Table S23 summarizes the task-specific GEE results. Several linguistic features showed significant associations with diagnosis after FDR correction. Average Closeness Centrality of the semantic graph, Adverb Ratio, Interjection Ratio, and Case Particle (Kakujoshi) Ratio all showed significant negative associations with schizophrenia status. Age reached marginal significance, whereas gender and most other syntactic or lexical features did not show significant effects.

| **Variable** | **Beta** | **SE** | **z** | **95%CI** | **OR** | **OR_CI** | **p** | **FDR_p** |
| --- | --- | --- | --- | --- | --- | --- | --- | --- |
| **Age** | **-0.46** | **0.21** | **-2.20** | **[-0.872 – -0.050]** | **0.63** | **[0.418 – 0.951]** | **0.028** | **0.062** |
| **Gender (female=1)** | **-0.63** | **0.42** | **-1.47** | **[-1.456 – 0.206]** | **0.54** | **[0.233 – 1.229]** | **0.141** | **0.223** |
| **Average Sentence Length** | **-0.50** | **0.24** | **-2.08** | **[-0.965 – -0.029]** | **0.61** | **[0.381 – 0.972]** | **0.038** | **0.070** |
| **Redundancy** | **0.12** | **0.20** | **0.60** | **[-0.269 – 0.507]** | **1.13** | **[0.764 – 1.661]** | **0.548** | **0.670** |
| **Average Closeness Centrality of Semantic Graph** | **-0.53** | **0.19** | **-2.73** | **[-0.909 – -0.150]** | **0.59** | **[0.403 – 0.861]** | **0.006** | **0.016** |
| **Adverb Ratio** | **-0.61** | **0.20** | **-3.01** | **[-1.001 – -0.212]** | **0.55** | **[0.368 – 0.809]** | **0.003** | **0.011** |
| **Interjection Ratio** | **-0.58** | **0.19** | **-3.09** | **[-0.948 – -0.213]** | **0.56** | **[0.388 – 0.808]** | **0.002** | **0.011** |
| **Numeral Ratio** | **-0.01** | **0.20** | **-0.03** | **[-0.394 – 0.381]** | **0.99** | **[0.674 – 1.464]** | **0.975** | **0.975** |
| **Determiner Ratio** | **-0.27** | **0.20** | **-1.40** | **[-0.655 – 0.110]** | **0.76** | **[0.519 – 1.116]** | **0.162** | **0.223** |
| **Case Particle (Kakujoshi) Ratio** | **-1.16** | **0.25** | **-4.57** | **[-1.657 – -0.662]** | **0.31** | **[0.191 – 0.516]** | **<0.001** | **<0.001** |
| **Named Entity (PERSON) Ratio** | **0.09** | **0.19** | **0.48** | **[-0.276 – 0.456]** | **1.09** | **[0.759 – 1.578]** | **0.630** | **0.693** |
| **Constant** | **0.78** | **0.29** | **2.70** | **[0.214 – 1.350]** | **2.19** | **[1.238 – 3.857]** | **0.007** |  |

Table S23. Task 3 GEE results using representative linguistic features

| **Metric** | **Estimate** | **95% CI (Lower)** | **95% CI (Upper)** |
| --- | --- | --- | --- |
| AUC | 0.867 | 0.745 | 0.959 |
| Sensitivity | 0.762 | 0.568 | 0.944 |
| Specificity | 0.684 | 0.474 | 0.882 |
| PPV | 0.727 | 0.529 | 0.905 |
| NPV | 0.722 | 0.5 | 0.929 |
| Accuracy | 0.725 | 0.575 | 0.85 |
| F1 | 0.744 | 0.563 | 0.875 |
| Balanced Accuracy | 0.723 | 0.58 | 0.854 |

Table S24. Predictive Performance of the Task 3 Classifier

**S 2-5. Sensitivity Analysis: GEE Model Using Factor Scores**

In Tasks 1 and 3, the factor-score GEE models and the representative-feature GEE models identified the same factors as significant. In Task 2, six factor scores reached significance, whereas four representative features were significant. Importantly, all factors with significant representative features were also significant in the factor-score model.
This modest discrepancy reflects that factor scores capture variance shared across multiple features, whereas representative features reflect only a single dominant variable. Consequently, some Task 2 factors with moderately distributed loadings were detectable only through factor scores. This pattern is expected and does not alter the substantive interpretation of the diagnostic associations.

| **Variable** | **Odds Ratio** | **Std. Error** | **z** | **P>\|z\|** | **95% CI Lower (OR)** | **95% CI Upper (OR)** |
| --- | --- | --- | --- | --- | --- | --- |
| age | 0.79 | 0.25 | -0.94 | 0.35 | 0.48 | 1.30 |
| 2.gender | 0.24 | 0.48 | -2.97 | < 0.001 | 0.09 | 0.62 |
| factor1 | 1.22 | 0.23 | 0.88 | 0.38 | 0.78 | 1.92 |
| factor2 | 0.15 | 0.29 | -6.40 | < 0.001 | 0.09 | 0.27 |
| factor3 | 0.68 | 0.19 | -1.93 | 0.05 | 0.47 | 1.01 |
| factor4 | 0.90 | 0.23 | -0.42 | 0.67 | 0.57 | 1.43 |
| factor5 | 0.84 | 0.26 | -0.65 | 0.51 | 0.51 | 1.40 |
| factor6 | 3.35 | 0.25 | 4.93 | < 0.001 | 2.08 | 5.42 |
| factor7 | 2.14 | 0.23 | 3.28 | < 0.001 | 1.36 | 3.39 |
| factor8 | 1.38 | 0.22 | 1.44 | 0.15 | 0.90 | 2.12 |
| factor9 | 1.21 | 0.19 | 1.02 | 0.31 | 0.84 | 1.75 |

Table S25. Task 1 GEE results using factor scores

| **Variable** | **Odds Ratio** | **Std. Error** | **z** | **P>\|z\|** | **95% CI Lower (OR)** | **95% CI Upper (OR)** |
| --- | --- | --- | --- | --- | --- | --- |
| age_z | 0.75 | 0.23 | -1.26 | 0.21 | 0.48 | 1.17 |
| 2.gender | 0.51 | 0.42 | -1.60 | 0.11 | 0.22 | 1.16 |
| factor1 | 2.05 | 0.20 | 3.66 | < 0.001 | 1.40 | 3.02 |
| factor2 | 1.58 | 0.18 | 2.53 | 0.01 | 1.11 | 2.25 |
| factor3 | 1.25 | 0.17 | 1.36 | 0.17 | 0.91 | 1.73 |
| factor4 | 1.54 | 0.19 | 2.31 | 0.02 | 1.07 | 2.22 |
| factor5 | 0.89 | 0.18 | -0.65 | 0.51 | 0.63 | 1.26 |
| factor6 | 0.60 | 0.22 | -2.33 | 0.02 | 0.39 | 0.92 |
| factor7 | 4.72 | 0.39 | 4.02 | < 0.001 | 2.21 | 10.04 |
| factor8 | 0.91 | 0.16 | -0.62 | 0.54 | 0.66 | 1.24 |
| factor9 | 0.51 | 0.23 | -2.90 | < 0.001 | 0.32 | 0.80 |
| factor10 | 0.71 | 0.21 | -1.69 | 0.09 | 0.47 | 1.06 |
| factor11 | 1.11 | 0.20 | 0.52 | 0.60 | 0.76 | 1.63 |

Table S26. Task 2 GEE results using factor scores

| **Variable** | **Odds Ratio** | **Std. Error** | **z** | **P>\|z\|** | **95% CI Lower (OR)** | **95% CI Upper (OR)** |
| --- | --- | --- | --- | --- | --- | --- |
| age | 0.59 | 0.24 | -2.20 | 0.03 | 0.37 | 0.94 |
| 2.gender | 0.53 | 0.43 | -1.48 | 0.14 | 0.23 | 1.23 |
| factor1 | 0.71 | 0.19 | -1.74 | 0.08 | 0.49 | 1.04 |
| factor2 | 0.73 | 0.20 | -1.60 | 0.11 | 0.50 | 1.07 |
| factor3 | 1.09 | 0.20 | 0.42 | 0.67 | 0.74 | 1.60 |
| factor4 | 2.40 | 0.30 | 2.92 | < 0.01 | 1.33 | 4.33 |
| factor5 | 0.53 | 0.21 | -3.03 | < 0.01 | 0.35 | 0.80 |
| factor6 | 0.30 | 0.23 | -5.17 | < 0.001 | 0.19 | 0.48 |
| factor7 | 1.37 | 0.24 | 1.34 | 0.18 | 0.86 | 2.18 |
| factor8 | 0.37 | 0.21 | -4.66 | < 0.001 | 0.24 | 0.56 |
| factor9 | 1.40 | 0.21 | 1.62 | 0.11 | 0.93 | 2.10 |

Table S27. Task 3 GEE results using factor scores

**S 2-6. Sensitivity Analysis: Task 1-specific GEE Model Adjusted for Years of Education**

To assess the robustness of the primary GEE model, we conducted a sensitivity analysis in which years of education was included as an additional covariate. Participants with non-missing education data were included, resulting in 99 participants in the training set and 22 in the test set (SCZ = 51, HC = 70 in total). Table S28 summarizes the demographic characteristics of the training and test partitions, and Table S29 presents the GEE coefficients adjusted for years of education.

The results showed that the main linguistic predictors (e.g., mean pairwise word similarity, adverb ratio, case particles) remained significant even after controlling for education. Years of education was a significant covariate (β = −0.92, p = 0.011), indicating that lower education level was associated with higher odds of SCZ. Overall, the inclusion of education did not materially alter the interpretation of the primary results.

| Variable | Train | Test |
| --- | --- | --- |
| Participants (N) | 99 | 22 |
| SCZ (n) | 45 | 6 |
| HC (n) | 54 | 16 |
| Age (mean ± SD) | 51.74 ± 16.73 | 54.23 ± 18.41 |
| Years of Education (mean ± SD) | 14.46 ± 2.96 | 14.89 ± 2.09 |
| Male (n) | 47 | 10 |
| Female (n) | 52 | 12 |
| Visits (unique count) | 5 | 4 |

Table S28. Demographic Characteristics of Training and Test Sets (Education Non-Missing Subsample)

Values represent mean ± SD unless otherwise indicated. N = number of unique participants.

| **Variable** | **Beta** | **SE** | **z** | **95%CI** | **OR** | **OR_CI** | **p** | **FDR_p** |
| --- | --- | --- | --- | --- | --- | --- | --- | --- |
| Age | -0.75 | 0.34 | -2.17 | [-1.423 – -0.072] | 0.47 | [0.241 – 0.930] | 0.03 | 0.06 |
| Years of Education | -0.92 | 0.36 | -2.55 | [-1.629 – -0.213] | 0.4 | [0.196 – 0.808] | 0.011 | 0.026 |
| Gender (female=1) | -1.77 | 0.64 | -2.77 | [-3.018 – -0.518] | 0.17 | [0.049 – 0.596] | <0.01 | 0.018 |
| Number of Sentences | 0.32 | 0.3 | 1.09 | [-0.258 – 0.902] | 1.38 | [0.773 – 2.464] | 0.277 | 0.332 |
| Mean Pairwise Word Similarity | 0.94 | 0.33 | 2.83 | [0.287 – 1.586] | 2.55 | [1.333 – 4.883] | <0.01 | 0.018 |
| Adverb Ratio | -1.26 | 0.43 | -2.95 | [-2.103 – -0.425] | 0.28 | [0.122 – 0.654] | <0.01 | 0.018 |
| Interjection Ratio | 0.23 | 0.35 | 0.66 | [-0.452 – 0.908] | 1.26 | [0.636 – 2.479] | 0.511 | 0.557 |
| Case Particles (Kakujoshi) Ratio | -1.56 | 0.45 | -3.44 | [-2.447 – -0.670] | 0.21 | [0.087 – 0.512] | <0.01 | 0.012 |
| Sentence-final Particles (Shuujoshi) Ratio | -0.67 | 0.38 | -1.76 | [-1.411 – 0.077] | 0.51 | [0.244 – 1.080] | 0.079 | 0.118 |
| Prenominal Adjective Ratio | 0.51 | 0.34 | 1.48 | [-0.164 – 1.175] | 1.66 | [0.849 – 3.240] | 0.139 | 0.185 |
| Nominal Modifier Ratio | 0.72 | 0.36 | 1.99 | [0.012 – 1.431] | 2.06 | [1.012 – 4.182] | 0.046 | 0.079 |
| Tree Height Ratio | -0.15 | 0.3 | -0.49 | [-0.731 – 0.440] | 0.86 | [0.481 – 1.552] | 0.626 | 0.626 |
| Constant | 1.34 | 0.47 | 2.83 | [0.409 – 2.262] | 3.8 | [1.506 – 9.601] | <0.01 | - |

Table S29. Sensitivity Analysis: Task 1-specific GEE Model Adjusted for Years of Education

Coefficients are from a population-averaged GEE logistic model predicting diagnosis (SCZ vs HC), adjusted for age, gender, years of education, and linguistic features. OR = odds ratio; CI = confidence interval; FDR_p = Benjamini–Hochberg corrected p-values.

**S2-6. Cross-task Robustness**

*Cross-task consistency (PC results).*

The PC analysis identified two features—Adverb Ratio (q_PC_ = 0.021) and Case Particles (Kakujoshi) Ratio (q_PC_ = 0.014)—that showed significant cross-task consistency (u = 2 of 3 tasks). Other features, such as Age, Gender, Interjection Ratio, and Redundancy, did not reach significance after FDR correction.

| **Feature** | **p(Task1)** | **p(Task2)** | **p(Task3)** | **M(Tasks)** | **p_(2)_** | **p_PC_** | **q_PC_ (BH)** | **Direction consistency** |
| --- | --- | --- | --- | --- | --- | --- | --- | --- |
| Case Particles (Kakujoshi) Ratio | <0.001 | - | <0.001 | 2 | < 0.01 | < 0.01 | 0.01 | Yes (−, −) |
| Adverb Ratio | <0.001 | - | 0.00 | 2 | < 0.01 | 0.01 | 0.02 | Yes (−, −) |
| Age | 0.66 | 0.25 | 0.03 | 3 | 0.25 | 0.76 | 1.00 | Yes (−, −, −) |
| Gender (female = 1) | 0.05 | 0.13 | 0.14 | 3 | 0.13 | 0.39 | 0.92 | Yes (−, −, −) |
| Interjection Ratio | 0.53 | - | 0.00 | 2 | 0.53 | 1.00 | 1.00 | Yes (−, −) |
| Redundancy | - | 0.02 | 0.55 | 2 | 0.55 | 1.00 | 1.00 | Mixed (−, ＋) |
| Numeral Ratio | - | 0.36 | 0.98 | 2 | 0.98 | 1.00 | 1.00 | Mixed (＋, −) |

**Table S30. Partial-Conjunction Results for Cross-Task Consistency of Linguistic Features**
This table summarizes the partial-conjunction (PC) analysis assessing whether each linguistic feature shows statistically reliable associations with diagnosis across multiple tasks. Columns list task-specific p-values, the number of tasks showing non-null effects (M), the PC p-value (p(2)), its corresponding partial-conjunction p-value with Benjamini–Hochberg correction (qPC), and the consistency of effect directions. Significant cross-task features—Adverb Ratio and Case Particles (Kakujoshi) Ratio—correspond to those reported in the main text.

*Task × feature GEE sensitivity analysis.*

GEE models were fitted to the two PC-positive features (Adverb Ratio and Case Particles (Kakujoshi) Ratio). Results are summarized below. Adverb Ratio showed a strong negative association with schizophrenia in Task 1, with significant interactions indicating attenuation in Tasks 2–3. In contrast, the effect of Case Particles (Kakujoshi) Ratio remained stable across all tasks with no significant interactions. Taken together, these results indicate that case-particle reduction is a task-general marker, whereas adverb reduction is strongest in spontaneous conversation but directionally consistent across tasks.

*Adverb Ratio*

| **Parameter** | **β** | **SE** | **z** | **p** | **OR [95% CI]** |
| --- | --- | --- | --- | --- | --- |
| Adverb Ratio | -0.92 | 0.19 | -4.92 | <0.001 | 0.40 [0.28–0.58] |
| Task 2 × Adverb Ratio | 0.81 | 0.18 | 4.58 | <0.001 | 2.25 [1.59–3.19] |
| Task 3 × Adverb Ratio | 0.92 | 0.2 | 4.71 | <0.001 | 2.52 [1.72–3.70] |
| age | -0.014 | 0.011 | -1.29 | 0.20 | — |
| gender (female=1) | -0.47 | 0.32 | -1.46 | 0.14 | — |

**Table S31. Task-by-Task Interaction Effects for Adverb Ratio**
This table reports the generalized estimating equation (GEE) model testing whether the association between Adverb Ratio and schizophrenia diagnosis differs across tasks. Adverb Ratio showed a strong negative association with diagnosis in Task 1, while significant positive interaction terms indicate that the effect was attenuated in Task 2 and Task 3. The interaction was statistically significant overall (Wald χ²(2) = 35.2, p < 0.001).

*Case Particles (Kakujoshi) Ratio*

| **Variable** | **β** | **SE** | **z** | **p** | **OR [95% CI]** |
| --- | --- | --- | --- | --- | --- |
| Case Particles (Kakujoshi) Ratio | -0.66 | 0.17 | -3.88 | <0.001 | 0.52 [0.37–0.72] |
| Task 2 × Case Particles (Kakujoshi) Ratio | 0.12 | 0.18 | 0.65 | 0.52 | 1.12 [0.79–1.60] |
| Task 3 × Case Particles (Kakujoshi) Ratio | -0.17 | 0.22 | -0.78 | 0.43 | 0.85 [0.55–1.29] |
| age | -0.017 | 0.011 | -1.55 | 0.12 | — |
| gender (female=1) | -0.17 | 0.33 | -0.52 | 0.60 | — |

**Table S32. Task-by-Task Interaction Effects for Case Particles (Kakujoshi) Ratio**
This table shows the GEE model assessing whether the association between the Case Particles (Kakujoshi) Ratio and schizophrenia diagnosis varies by task. The main effect was significantly negative, indicating reduced case-particle use in schizophrenia. Interaction terms with Task 2 and Task 3 were nonsignificant (Wald χ²(2) = 37.4, p_int_ > 0.40), demonstrating that this effect was stable across all tasks.

**S 2-7. Multicollinearity Diagnostics**

To assess potential multicollinearity among predictors included in the task-specific GEE models, variance inflation factors (VIFs) were calculated using ordinary least squares (OLS) regression models including the same sets of predictors as each GEE analysis. Consistent with our prespecified criterion, predictors with VIF > 10 were considered indicative of substantial redundancy.

Across all three task-specific models, VIF values were uniformly low. No predictor exceeded a VIF of 3 in any model, indicating minimal multicollinearity and supporting the stability of parameter estimates in the reported GEE analyses.

| **Task** | **Variable** | **VIF** | **1 over VIF** |
| --- | --- | --- | --- |
| **1** | Age | 1.46 | 0.68 |
| **1** | Gender | 1.37 | 0.73 |
| **1** | Number of Sentences | 1.36 | 0.73 |
| **1** | Mean Pairwise Word Similarity | 1.39 | 0.72 |
| **1** | Adverb Ratio | 1.59 | 0.63 |
| **1** | Interjection Ratio | 1.8 | 0.55 |
| **1** | Case Particle (Kakujoshi) Ratio | 2.35 | 0.43 |
| **1** | Sentence-final Particles (Shuujoshi) Ratio | 1.59 | 0.63 |
| **1** | Prenominal Adjective Ratio | 1.35 | 0.74 |
| **1** | Nominal Modifier Ratio | 1.71 | 0.58 |
| **1** | Normalized Tree Height | 1.39 | 0.72 |
| **1** | Mean VIF | 1.58 |  |

**Table S33. Variance Inflation Factors (VIFs) for Predictors Included in the Task 1 GEE Model**

Variance inflation factors were calculated using an OLS model including the same predictors as the Task 1 GEE analysis. All VIF values were below 3 (mean VIF = 1.58), indicating no evidence of problematic multicollinearity.

| **Task** | **Variable** | **VIF** | **1 over VIF** |
| --- | --- | --- | --- |
| **2** | Age | 1.13 | 0.88 |
| **2** | Gender (female = 1) | 1.1 | 0.91 |
| **2** | Redundancy | 1.12 | 0.90 |
| **2** | Density of Semantic Graph | 1.08 | 0.93 |
| **2** | Numeral Ratio | 1.08 | 0.93 |
| **2** | Auxiliary Verb Ratio | 1.27 | 0.79 |
| **2** | Subordinating Conjunction Ratio | 1.5 | 0.67 |
| **2** | Particle Ratio | 1.31 | 0.76 |
| **2** | Adverbial Modifier Ratio | 1.02 | 0.98 |
| **2** | Coordinating Conjunction Ratio | 1.33 | 0.75 |
| **2** | Discourse Ratio | 1.63 | 0.61 |
| **2** | Global Perseveration Ratio | 1.18 | 0.84 |
| **2** | Normalized Dependency Distance | 1.35 | 0.74 |
| **2** | Mean VIF | 1.24 |  |

**Table 34. Variance Inflation Factors (VIFs) for Predictors Included in the Task 2 GEE Model**

Variance inflation factors were calculated using an OLS model including the same predictors as the Task 2 GEE analysis. All VIF values were below 2 (mean VIF = 1.24), indicating excellent model stability.

| **Task** | **Variable** | **VIF** | **1 over VIF** |
| --- | --- | --- | --- |
| **3** | Age | 1.23 | 0.81 |
| **3** | Gender (female=1) | 1.12 | 0.89 |
| **3** | Average Sentence Length | 1.35 | 0.74 |
| **3** | Redundancy | 1.18 | 0.85 |
| **3** | Average Closeness Centrality of Semantic Graph | 1.2 | 0.83 |
| **3** | Adverb Ratio | 1.46 | 0.68 |
| **3** | Interjection Ratio | 1.11 | 0.90 |
| **3** | Numeral Ratio | 1.12 | 0.89 |
| **3** | Determiner Ratio | 1.1 | 0.91 |
| **3** | Case Particle (Kakujoshi) Ratio | 1.8 | 0.56 |
| **3** | Named Entity (PERSON) Ratio | 1.04 | 0.96 |
| **3** | Mean VIF | 1.25 |  |

**Table S35. Variance Inflation Factors (VIFs) for Predictors Included in the Task 3 GEE Model**

Variance inflation factors were calculated using an OLS model including the same predictors as the Task 3 GEE analysis. All VIF values were below 2 (mean VIF = 1.25), supporting the absence of meaningful multicollinearity.

**Supplementary Reference**

Benjamini, Y., & Heller, R. (2008). Screening for partial conjunction hypotheses. *Biometrics, 64*(4), 1215–1222. <https://doi.org/10.1111/j.1541-0420.2007.00984.x>

Honnibal, M., & Montani, I. (2017). *spaCy 2: Natural language understanding with Bloom embeddings, convolutional neural networks and incremental parsing* [Computer software]. Explosion. <https://explosion.ai>

Kishimoto, T., Nakamura, H., Kano, Y., Eguchi, Y., Kitazawa, M., Liang, K. C., Kudo, K., Sento, A., Takamiya, A., Horigome, T., Yamasaki, T., Sunami, Y., Kikuchi, T., Nakajima, K., Tomita, M., Bun, S., Momota, Y., Sawada, K., Murakami, J., Takahashi, H., … Mimura, M. (2022). Understanding psychiatric illness through natural language processing (UNDERPIN): Rationale, design, and methodology. *Frontiers in Psychiatry, 13*, 954703. <https://doi.org/10.3389/fpsyt.2022.954703>

Koiso, H., Amatani, H., Den, Y., Iseki, Y., Ishimoto, Y., Kashino, W., … Watanabe, Y. (2022). Design and evaluation of the Corpus of Everyday Japanese Conversation. In *Proceedings of the Thirteenth Language Resources and Evaluation Conference (LREC 2022)* (pp. 5587–5594). European Language Resources Association.

Kudo, T., Yamamoto, K., & Matsumoto, Y. (2004). Applying conditional random fields to Japanese morphological analysis. In *Proceedings of the 2004 Conference on Empirical Methods in Natural Language Processing* (pp. 230–237).

Megagon Labs. (2023). *GiNZA Japanese NLP library* [Computer software]. <https://github.com/megagonlabs/ginza>

Mikolov, T., Chen, K., Corrado, G., & Dean, J. (2013). Efficient estimation of word representations in vector space. In *Proceedings of the International Conference on Learning Representations*. <https://arxiv.org/abs/1301.3781>

Omura, M., Matsuda, H., Asahara, M., & Wakasa, A. (2022). *UD_Japanese-CEJC: Dependency relation annotation on corpus of everyday Japanese conversation* [Poster presentation]. SIGDAT Meeting, Association for Computational Linguistics.

Salton, G., & Buckley, C. (1988). Term-weighting approaches in automatic text retrieval. *Information Processing & Management, 24*(5), 513–523. <https://doi.org/10.1016/0306-4573(88)90021-0>

Sasahara, K. (2016). Visualizing collective attention using association networks. *Transactions of the Japanese Society for Artificial Intelligence*. Advance online publication. <https://doi.org/10.1527/tjsai.B-MDF02>

Sato, T., Hashimoto, T., & Okumura, M. (2017). Implementation of a word segmentation dictionary called mecab-ipadic-NEologd and study on how to use it effectively for information retrieval (in Japanese). In *Proceedings of the Twenty-Third Annual Meeting of the Association for Natural Language Processing*.

Sonoisa. (2020). *Sentence-BERT Japanese (base, mean-tokens)* [Computer software]. <https://huggingface.co/sonoisa/sentence-bert-base-ja-mean-tokens>

Suzuki, M. (2018). *WikiEntVec: Japanese Word2Vec model trained on Wikipedia* [Computer software]. <https://github.com/singletongue/WikiEntVec>

Suzuki, M., & Nagata, M. (2018). Sudachi: A Japanese tokenizer for precision and flexibility. In *Proceedings of the Workshop on Open-Source Arabic Corpora and Processing Tools*.

Toto, S., Murata, M., Tokuhisa, M., & Ma, Q. (2014). Detecting redundant sentences using machine learning and redundancy. In *Proceedings of the 20th Annual Meeting of the Association for Natural Language Processing* (pp. 939–942).

Works Applications. (2023). *SudachiDict-core: Dictionary for Sudachi Japanese tokenizer* [Computer software]. <https://github.com/WorksApplications/SudachiDict>
